# A Cox Rate-and-State Model for Monitoring Seismic Hazard in the Groningen Gas Field

**DOI:** 10.1007/s11004-025-10222-4

**Published:** 2025-08-19

**Authors:** Z. Baki, M. N. M. van Lieshout

**Affiliations:** 1 Credit Risk Management of Digital Products, Halyk Bank JSC, Al-Farabi 40, Almaty, Kazakhstan; 2 Centrum Wiskunde and Informatica (CWI), P.O. Box 94079, NL-1090 GB Amsterdam, The Netherlands; 3 Faculty of Electrical Engineering, Mathematics & Computer Science, University of Twente, P.O. Box 217, NL-7500 AE Enschede, The Netherlands

**Keywords:** Cox process, Gas production, Induced seismicity, Pore pressure, Rate-and-state model, Spatio-temporal point process, 60G55, 62F15, 62M30

## Abstract

To monitor the seismic hazard in the Groningen gas field, this paper modifies the rate-and-state model that relates changes in pore pressure to induced seismic hazard by allowing for noise in pore pressure measurements and by explicitly taking into account gas production volumes. The first and second-moment structures of the resulting Cox process are analysed, an unbiased estimating equation approach for the unknown model parameters is proposed and the conditional distribution of the driving random measure is derived. A parallel Metropolis-adjusted Langevin algorithm is used for sampling from the conditional distribution and to monitor the hazard.

## Introduction

The study of induced earthquakes caused by the extraction or injection of fluids or gases is an important research topic. In the Netherlands, the Groningen gas field, discovered in the late 1950s, has played an important role in the Dutch economy. With an estimated recoverable gas volume of over 2,900 billion normal cubic metres spread over a region of about 900 square kilometres, it is one of the largest gas fields on the planet (Jager and Visser 2017). However, large production volumes in the 1970s caused a drop in pore pressure in the gas field which resulted in induced earthquakes in the previously seismically inactive region. The severest tremor to date occurred in August 2012 and had a magnitude of 3.6. It is estimated that the maximum possible induced magnitude is around 4 (Boitz et al. 2024). Thus, it is essential to be able to predict seismic hazard based on field measurements, for instance of pore pressure or, equivalently, Coulomb stress. One of the most widely used methodologies to do so is the rate-and-state model (Candela et al. 2019; Dempsey and Suckale 2017; Richter et al. 2020), which is now considered to be the state-of-the-art technique (Kűhn et al. 2022).

In the rate-and-state model, the earthquakes follow a Poisson point process whose intensity function $\lambda$ (the rate) is assumed to be inversely proportional to a state variable $\varGamma$ that is defined by an ordinary differential equation. This differential equation is based on physical considerations and takes into account the elapsed time and the change in pore pressure. Nevertheless, it can be criticised on several points. Firstly, since, by definition, the points in any Poisson point process do not interact with one another, the model is unable to deal with clustering as seen in, for instance, the Groningen data (Lieshout and Baki 2024). Secondly, the pressure values are assumed to be known everywhere. Lastly, the varying gas extraction is not taken into account. To address these shortcomings, in this paper, a doubly stochastic or Cox rate-and-state model is proposed. Additionally, a toolbox for statistical inference is developed.

The plan is as follows. First, Sect. 2 describes the data and reviews the Poisson rate-and-state model. Section 3 proposes a modified Cox rate-and-state model. Explicit expressions for the first and second moments of the state variable are given in Sect. 4 and, in Sect. 5, the delta method is applied to approximate the first two moments of the rate variable. Section 6 turns to the estimation of the model parameters. Next, Sect. 7 focuses on the random state variable. Its conditional distribution given earthquake count data is calculated and a parallel Metropolis-adjusted Langevin monitoring algorithm is applied to the Groningen data. The paper closes with a discussion and some suggestions for future research.

## Data

In a previous paper, Lieshout and Baki (2024) carried out an exhaustive exploratory analysis of the Groningen earthquake data, including data collection, cleaning and smoothing. Here, the procedure is summarised briefly; the reader is referred to the previous work for full details.

### The Groningen Gas Field

**Fig. 1:**
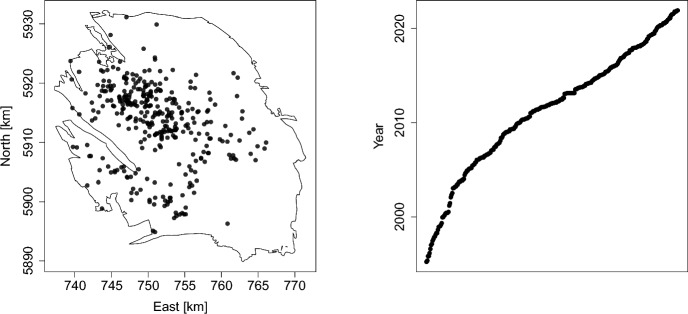
Spatial (left-most panel) and temporal (right-most panel) representations of the 332 earthquakes of magnitude 1.5 or larger with epicentre in the Groningen gas field that occurred in the period from January 1st, 1995, up to December 31st, 2021. The spatial coordinates are in the UTM-31 system with kilometres as unit. The dates are given in years

The Royal Dutch Meteorological Office (KNMI) maintain an earthquake catalogue for the Netherlands (KNMI 2022). Here, we restrict attention to those tremors that occur within the Groningen gas field in the north of the country. Figure 1 shows a spatial and temporal visualisation for this field of the 332 earthquakes of magnitude at least 1.5, the magnitude of completeness, in the period from January 1st, 1995, up to December 31st, 2021. Since all earthquakes occur at the same depth, a two-dimensional analysis suffices.

The left panel is based on shapefiles from the Geological Survey of the Netherlands (TNO 2022) website. The files are updated monthly and contain a polygonal approximation of the border of the field. The map that was published in April 2022 is used here. The coordinates of the field are given in the UTM system using zone 31 with metres as the spatial unit, which in this paper are rescaled to kilometres. Note that earthquakes seem to happen more often in the central and southwestern parts of the gas field.

The right panel shows the time of occurrence of each earthquake against their chronological index number. The steeper curve in the 1990s reflects the longer spell between successive earthquake occurrences; flatter pieces indicate a quicker succession of earthquakes.

**Fig. 2:**
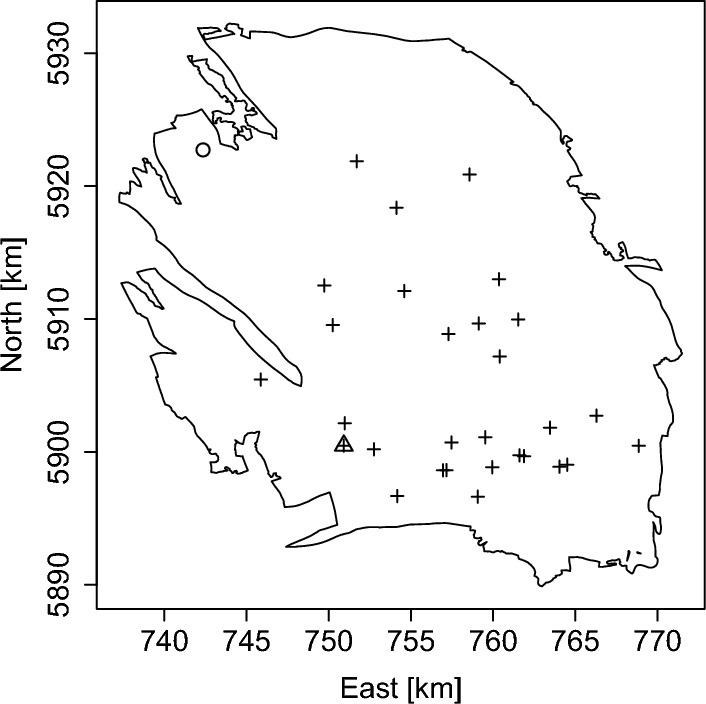
The crosses represent the locations in the UTM-31 coordinate system (in kilometres) of 29 production wells in the Groningen gas field. The one marked by a triangle is ‘Slochteren’. Also shown is an observation well at Oldorp indicated by a circle

To explain the observed heterogeneity, two covariates are available. Monthly gas production values from the start of preliminary exploration in February 1956 up to and including December 2021 for all 29 production wells present in the field, indicated by a cross in Fig. 2, were kindly provided to the authors.^1^ The production is recorded in cubic metres, which is here rescaled to billion cubic metres (Nbcm).

In order to be useful as covariate information in statistical models, the raw data over space and time are smoothed by means of an adaptive kernel approach. In doing so, values are obtained for every location and time point, which can then be aggregated as desired.

**Fig. 3:**
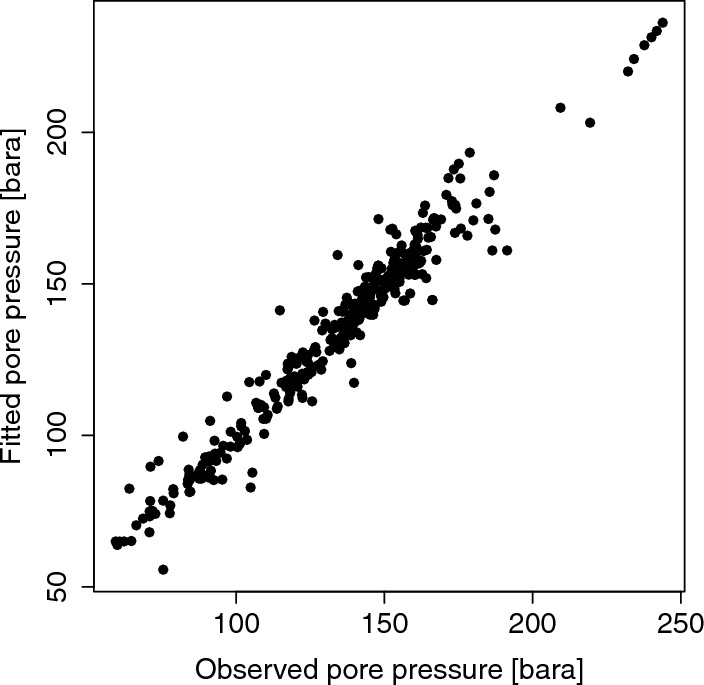
Fitted pore pressure against observed values in bara from a fourth-order polynomial Gaussian regression model (Appendix A)

Additionally, 2, 009 pore pressure observations in bara, the unit for absolute pressure, are available on the website of the Nederlandse Aardolie Maatschappij (NAM), the exploration and production company, over the period from April 1960 until November 2018 (NAM 2022). Of these, only 352 measurements fall within the time frame used in this paper. To be able to assign a pore pressure value to each spatial location and every point in time, Lieshout and Baki (2024) fitted a polynomial regression model by the least squares method to the 352 measurements whose temporal coordinates lie in 1995 or in later years. The analysis of variance (ANOVA) table, the estimated regression parameters and their $95\%$ confidence, under the assumption that the error terms are independent Gaussians with zero mean, are given in Appendix A. The variance of the noise was estimated as well, and its estimate is also provided in Appendix A. To validate the model, Fig. 3 plots the fitted pore pressure against the observed values. Since the points lie approximately on a straight line, the fit is deemed adequate.

### The Poisson Rate-and-State Model

In the classic rate-and-state model (Candela et al. 2019; Dempsey and Suckale 2017), earthquakes occur according to a spatio-temporal Poisson point process. Such a process is completely specified by an intensity function $\lambda$ that describes the probability of an event occurring at a given time and location. For sets $W_S$ in some spatial observation window and $W_T$ in a temporal domain, the probability distribution of the number of events in $W_S \times W_T$ is a Poisson with rate $\int _{W_S \times W_T} \lambda (\textbf{s},t) d\textbf{s} dt$. Moreover, given that there are *n* events, these are scattered independently according to the probability density function $\lambda / \int _{W_S\times W_T} \lambda (\textbf{s},t) d\textbf{s} dt$. Thus, there are no interactions between the events.

The rate-and-state model is defined by

1 $$\begin{aligned} \lambda (\textbf{s},t) = r_0 \frac{\varGamma (\textbf{s},t_0)}{\varGamma (\textbf{s},t)}, \quad (\textbf{s},t) \in W_S \times W_T, \end{aligned}$$

where $W_S \subset {\mathbb R}^2$ is a compact subset of the plane, $W_T$ a closed and bounded interval in ${\mathbb R}$ and $t_0 = \min \{t: t \in W_T \}$. The parameter $r_0 > 0$ is the background seismicity; the state variable $\varGamma (\textbf{s},t)$ is defined by the ordinary differential equation

2 $$\begin{aligned} d\varGamma (\textbf{s},t) = \alpha \left( dt + \varGamma (\textbf{s},t) dX(\textbf{s},t) \right) , \end{aligned}$$

where $\alpha > 0$ is a model parameter and $X(\textbf{s},t)$ is the pore pressure at spatial location $\textbf{s}$ and time *t*. In the classic model, $X: W_S\times W_T \rightarrow {\mathbb R}^+$ is a deterministic function. Note that when the pore pressure remains constant, the state variable increases over time and the earthquake hazard decreases according to Omori’s law (Utsu et al. 1995). When the pore pressure changes due to gas extraction or fluid injection, an increase in pore pressure due to fluid injection emphasises the temporal increase in the state variable and thus reduces the seismic hazard even more. A drop in pore pressure contributes a negative term to Eq. (2); its effect on the intensity of earthquakes also depends on the temporal term.

Multiplying both sides of Eq. (2) by $\exp ( -\alpha X(\textbf{s},t) )$, discretising in time steps of length $\varDelta > 0$ and writing $t_k = t_0 + k\varDelta$, $k=1, \dots , m$, one obtains the Euler difference equation

3 $$\begin{aligned} \varGamma (\textbf{s}, t_{k +1} ) = \left( \varGamma (\textbf{s}, t_k) + \alpha \varDelta \right) \exp \left( \alpha ( X(\textbf{s},t_{k+1} ) - X(\textbf{s},t_k) ) \right) , \quad \textbf{s} \in W_S. \end{aligned}$$

The parameters $r_0$, $\alpha$ and the initial state $\varGamma (\textbf{s}, t_0) \equiv \gamma _0 > 0$ are treated as unknowns and can be estimated, for example, by the maximum likelihood method. For a full discussion and comparison with other techniques, see Kűhn et al. (2022). In line with current practice, the spatial domain $W_S$ is discretised in a regular grid with cell representatives $\textbf{s}_1, \dots , \textbf{s}_n$. Note that because $W_S$ is not necessarily rectangular, grid cells may have different areas, which are denoted by $\varDelta (\textbf{s}_i)$. For our data, take $\varDelta$ equal to 1 year and partition $W_S$ in a 32 by 32 grid.

The model defined by Eq. (1), Eq. (2) and Eq. (3) has several shortcomings. First of all, of the two covariates at our disposal, only one is used. Secondly, an exploratory analysis in Lieshout and Baki (2024) indicated that the data exhibit clustering that cannot be accounted for by the Poisson model. Finally, the variance of the regression model for the pore pressure is ignored, but should be taken into account given the sparsity of observations. In the next section, the rate-and-state model will be modified so that these issues are resolved.

## The Cox Rate-and-State Model

The Poisson rate-and-state model discussed in Sect. 2.2 expresses the change in seismic hazard in terms of elapsed time and pore pressure change. In practice, the values of the latter are typically available only at wells and monitoring stations. At other locations, the pore pressure must be estimated (Lieshout and Baki 2024) or approximated by linear (or spline-based) interpolation (Richter et al. 2020). For the Groningen data described in Sect. 2, there are only about 2,000 observations scattered unevenly over the field and spanning a period of several decades. Therefore, it would be better to explicitly take the uncertainty into account and treat the $X(\textbf{s}_i, t_j)$ as random variables. By doing so, one obtains a doubly stochastic or Cox point process (Chiu et al. 2013). Briefly, given a realisation $\lambda$ of the density $\varLambda$ of a random measure on $W_S\times W_T$, the driving random measure of the Cox process, the earthquakes form a Poisson point process with intensity function $\lambda$. Thus, the distribution of the Cox process is fully characterised by the distribution of the driving random measure.

To find a suitable driving random measure, assume that the pore pressure can be decomposed into a deterministic and stochastic part, that is,

4 $$\begin{aligned} X(\textbf{s}_i, t_j) = m(\textbf{s}_i, t_j) + E(\textbf{s}_i, t_j). \end{aligned}$$

Here $E(\textbf{s}_i,t_j)$ are independent mean-zero random variables with variance $\sigma ^2$ and *m* is the fitted polynomial regression model described in Sect. 2 and Appendix A. Since the variogram of the residuals is flat in time (Lieshout and Baki 2024), the residual variation is a function of spatial location only, which, since the rate-state equation (cf. Eq. (2)) depends only on temporal changes in pore pressure, may be ignored.

The random variables $X(\textbf{s}_i, t_j)$ are then plugged into the Euler difference equation (cf. Eq. (3)), and upon solving for the random variable $\varGamma (\textbf{s}_i, t_j)$, one obtains

5 $$\begin{aligned} \varGamma (\textbf{s}_i, t_j) = \exp \left( \alpha X(\textbf{s}_i, t_j) \right) \left\{ \alpha \varDelta \sum _{k=0}^{j-1} \exp \left( - \alpha X(\textbf{s}_i, t_k) \right) + \gamma _0 \exp \left( - \alpha X(\textbf{s}_i, t_0) \right) \right\} . \end{aligned}$$

The interpretation of the parameter $\alpha$ is the same as for the original rate-and-state model. In particular, when the pore pressure decreases as a consequence of gas extraction, $\alpha$ must be positive. As for the classic model, $\gamma _0>0$ is the initial state of the stochastic difference equation.

The other explanatory variable at our disposal is the vector of smoothed gas production (cf. Sect. 2). Its influence on the earthquake intensity function can be modelled through the multiplier $r_0$ in Eq. (1). Specifically, set $\varDelta$ equal to 1 year. This choice of $\varDelta$ is convenient, as it implies that there is no need to account for seasonal fluctuations. Also, write $V(\textbf{s}_i, t_j)$ for the volume of gas extracted in the grid cell around $\textbf{s}_i \in W_S$ over the year preceding $t_j$. The idea is then to replace the constant $r_0$ by $\exp \left( \theta _1 + \theta _2 V(\textbf{s}_i, t_j) \right)$. The real-valued parameter $\theta _1$ is the intercept and the parameter $\theta _2$ quantifies the effect of gas extraction. For positive $\theta _2$, an increase in production tends to increase the number of earthquakes. Mathematically, the model is well-defined for $\theta _2 \le 0$ as well, but does not make practical sense.

In summary, one obtains a Cox process $\varPsi$ with driving random measure defined by its density function

6 $$\begin{aligned} \varLambda (\textbf{s}_i,t_j) = \exp \left( \theta _1 + \theta _2 V(\textbf{s}_i,t_j) \right) \frac{ \gamma _0}{ \varGamma (\textbf{s}_i,t_j)}. \end{aligned}$$

Thus, writing $N(\textbf{s}_i,t_j)$ for the number of earthquakes in the space-time cell defined by $\textbf{s}_i$ and $t_j$, conditional on $\varLambda (\textbf{s}_i, t_j)$, $N(\textbf{s}_i, t_j)$ is Poisson distributed with rate parameter $\varLambda (\textbf{s}_i,t_j) \varDelta (\textbf{s}_i) \varDelta$ independently of the earthquake counts in other cells. Following Richter et al. (2020), in the sequel Eq. (6) will be re-parametrised in terms of $\alpha$, $\theta _1$, $\theta _2$ and $\eta = \log (\alpha /\gamma _0)$.

The Cox model defined by Eq. (6) achieves the set goals: it incorporates both available covariates, accounts for the uncertainty in the pore pressure interpolation, and exhibits clustering in time within grid cells. The latter statement follows from the fact that the pair correlation function of the proposed Cox model is greater than 1, that is,

$$\begin{aligned} g( (\textbf{s}_i, t_j), (\textbf{s}_i, t_k) ) = e^{\alpha ^2\sigma ^2} \ge 1, \quad 0< t_j < t_k. \end{aligned}$$

Note that the greater the uncertainty, the stronger the clustering.

## Moments of the State Variable

Since the randomness in the driving random measure (cf. Eq. (6)) of our Cox process is induced by the state process $\varGamma$ through the error term *E* in Eq. (4), the first and second-moment properties of $\varGamma$ are investigated first.

Let $\varGamma$ be defined by Eq. (5) for some $\textbf{s}\in W_S$ and $t_j$, $j=0,\dots , m$. Set $d = \exp \left( \alpha ^2 \sigma ^2\right)$ and define, for $i,j \in \{ 0, \dots , m \}$, $f_{ij} = \exp \left( \alpha \left\{ m(\textbf{s}, t_i) - m(\textbf{s}, t_j) \right\} \right) .$ Then, for $k = 1, \dots , m$,

$$\begin{aligned} {\mathbb E}\left[ \varGamma (\textbf{s}, t_k)\right]= &  d \alpha \left( \varDelta \sum _{i=0}^{k-1} f_{ki} + e^{-\eta } f_{k0} \right) ,\\ \mathrm{{Var}}\left[ \varGamma (\textbf{s}, t_k) \right]= &  \alpha ^2 \varDelta ^2 d^2 (d^2 - 1) \sum _{i=0}^{k-1} f_{ki}^2 + \alpha ^2 \varDelta ^2 d^2 (d-1) \sum _{i=0}^{k-1} \sum _{i\ne j=0}^{k-1} f_{ki} f_{kj} \\ &  + 2 \alpha ^2 e^{-\eta } \varDelta d^2 f_{k0}^2 \left( d^2 - 1 + (d-1) \sum _{i=1}^{k-1} f_{0i} \right) \\ &  + \alpha ^2 e^{-2\eta } f_{k0}^2 d^2 (d^2 - 1), \end{aligned}$$

and, for $$0< k < l \le m$$,

7 $$\begin{aligned} \mathrm{{Cov}}( \varGamma (\textbf{s}, t_k), \varGamma (\textbf{s}, t_l) )= &  \alpha ^2 \varDelta ^2 d^2 \sum _{i=0}^{k-1} \left\{ f_{ki} f_{li} (d-1) - f_{li} \left( 1 - \frac{1}{d} \right) \right\} \nonumber \\ &  + \alpha ^2 e^{-\eta } ( 2 \varDelta + e^{-\eta }) d^2 f_{k0} f_{l0} ( d - 1) \nonumber \\ &  - \alpha ^2 e^{-\eta } \varDelta d^2 f_{l0} \left( 1 - \frac{1}{d} \right) . \end{aligned}$$

When fluid is injected into a field, the pore pressure typically increases. In this case, the state variables are positively correlated, that is, $\mathrm{{Cov}}( \varGamma (\textbf{s}, t_k), \varGamma (\textbf{s}, t_l) ) \ge 0$ for all *k*, *l*. When the pore pressure decreases, for example, due to gas extraction, the picture is more varied. If $\alpha \sigma ^2 > m(\textbf{s}, t_0)$, then $\mathrm{{Cov}}( \varGamma (\textbf{s}, t_k), \varGamma (\textbf{s}, t_l) ) \ge 0$ for all *k*, *l*. On the other hand, if $\alpha \sigma ^2 < \min _{i = 0, \dots , m-1} \left\{ m(\textbf{s}, t_i) - m(\textbf{s}, t_{i+1}) \right\} ,$ the minimal drop in pressure in between observation epochs, for all $0 \le k < l \le m$,

$$\begin{aligned} \mathrm{{Cov}}( \varGamma (\textbf{s}, t_k), \varGamma (s, t_l) ) - \alpha ^2 e^{-\eta } ( \varDelta + e^{-\eta } ) d^2 (d-1) f_{k0} f_{l0} \le 0. \end{aligned}$$

The proofs of these statements can be found in Appendix B. The following examples are illuminating.

**Table 1:** Estimated pore pressure in bara on January 1st in the years 1995–2021 near the town of Slochteren (UTM-31 coordinates (750, 5900) in kilometres), The Netherlands

Years							
1995–2001	179.81	177.39	174.86	172.20	169.42	166.50	163.48
2002–2008	160.32	157.05	153.65	150.13	146.49	142.72	138.82
2009–2015	134.81	130.68	126.43	122.04	117.53	112.91	108.16
2016–2021	103.28	98.29	93.17	87.94	82.56	77.08	

### Example 1

Table 1 lists estimated pore pressure values near the town of Slochteren, indicated by a triangle in Fig. 2, in the Groningen gas field in the Netherlands for January 1st, 1995–2021 (Lieshout and Baki 2024). Specifically, the first entry in the top row, 179.81 bara, corresponds to the year 1995, the second entry to 1996 and so on.

Note that the pore pressure values are decreasing due to gas extraction. Since the intensity of induced earthquakes was very low in 1995, $\gamma _0$ can be considered infinite. Hence $\eta = -\infty$ and Eq. (7) then implies that the covariance matrix of the random vector $\varGamma (\textbf{s}, t_k)_{k=1,\dots , m}$ has positive entries only.

### Example 2

Next, suppose that – in contrast to the previous example – the initial seismicity is very high, that is, $\gamma _0 = 0$ or $\eta = \infty$. Assume a linearly decreasing sequence of pore pressures $m(\textbf{s}, 0) = 3$, $m(\textbf{s}, 1) = 2$ and $m(\textbf{s}, 2) = 1$, and set $\alpha = 1$. Then the covariance matrix of the random vector $(\varGamma (\textbf{s}, 1), \varGamma (\textbf{s}, 2))$ is readily calculated. Indeed, $\mathrm{{Var}}[ \varGamma (\textbf{s}, 1)] = e^{-2} ( e^{4\sigma ^2} - e^{2 \sigma ^2})$ and $\mathrm{{Var}}[\varGamma (\textbf{s}, 2)] = (e^{-2} + e^{-4}) ( e^{4\sigma ^2} - e^{2\sigma ^2}) + 2 e^{-3} (e^{3\sigma ^2} - e^{2\sigma ^2})$. The off-diagonal entry,

$$\begin{aligned} \mathrm{{Cov}}( \varGamma (\textbf{s}, 1), \varGamma (\textbf{s}, 2) )= &  e^{2\sigma ^2} \left( e^{-3} ( e^{\sigma ^2} - 1 ) - e^{-2} ( 1 - e^{-\sigma ^2} ) \right) , \end{aligned}$$

is negative for $0< \sigma ^2 < 1$ and positive for $\sigma ^2 > 1$. For $\sigma ^2 \in \{ 0, 1 \}$, the random variables $\varGamma (\textbf{s}, 1)$ and $\varGamma (\textbf{s}, 2)$ are uncorrelated.

## Approximate Moments of the Rate Variable

Recall that in the Cox rate-and-state model defined in Sect. 3, the rate of induced earthquakes is inversely proportional to the state. Due to the form of Eq. (5), the moments of ${1} / {\varGamma (\textbf{s}, t_k)}$ are intractable, but one can use the delta method (Vaart 1998) to approximate them in terms of the tractable moments of $\varGamma (\textbf{s}, t_k)$. Indeed, for $\textbf{s}\in W_S$ and $k = 0, 1, \dots , m$,

8 $$\begin{aligned} {\mathbb E}\left[ \frac{1}{\varGamma (\textbf{s}, t_k)} \right] \approx \frac{1}{{\mathbb E}\left[ \varGamma (\textbf{s}, t_k) \right] } + \frac{\mathrm{{Var}}\left[ \varGamma (\textbf{s}, t_k ) \right] }{ ({\mathbb E}\left[ \varGamma (\textbf{s}, t_k) \right] )^3 } . \end{aligned}$$

Note that the approximation of the expectation of $\varGamma (\textbf{s}, t_k)^{-1}$ is at least as large as its ‘plug-in estimator’ $1/ {\mathbb E}\left[ \varGamma (\textbf{s}, t_k)\right]$.

The same approach can be used to obtain an approximation for the covariance. First, note that for $\textbf{s}\in W_S$ and $k, l = 0, 1, \dots , m$,

$$\begin{aligned} {\mathbb E}\left[ \frac{1}{\varGamma (\textbf{s}, t_k) \varGamma (\textbf{s}, t_l)} \right]\approx &  \frac{1}{{\mathbb E}\left[ \varGamma (\textbf{s}, t_k) \right] \, {\mathbb E}\left[ \varGamma (\textbf{s}, t_l) \right] } + \frac{ \mathrm{{Cov}}( \varGamma (\textbf{s}, t_k ), \varGamma (\textbf{s}, t_l ) ) }{ ({\mathbb E}\left[ \varGamma (\textbf{s}, t_k)\right] )^2 ({\mathbb E}\left[ \varGamma (\textbf{s}, t_l) \right] )^2} \\ &  + \frac{\mathrm{{Var}}\left[ \varGamma (\textbf{s}, t_k ) \right] }{ {\mathbb E}\left[ \varGamma (\textbf{s}, t_l ) \right] ({\mathbb E}\left[ \varGamma (\textbf{s}, t_k) \right] )^3} + \frac{\mathrm{{Var}}\left[ \varGamma (\textbf{s}, t_l ) \right] }{ {\mathbb E}\left[ \varGamma (\textbf{s}, t_k ) \right] ({\mathbb E}\left[ \varGamma (\textbf{s}, t_l) \right] )^3}. \end{aligned}$$

For the variance, subtract the product of ${\mathbb E}\left[ 1/\varGamma (\textbf{s},t_k)\right]$ and ${\mathbb E}\left[ 1/\varGamma (\textbf{s},t_l)\right]$. Upon approximation by the right-hand side of Eq. (8),

9 $$\begin{aligned} \mathrm{{Var}}\left[ \frac{1}{\varGamma (\textbf{s}, t_k)} \right] \approx \frac{ \mathrm{{Var}}\left[ \varGamma (\textbf{s}, t_k ) \right] }{ ({\mathbb E}\left[ \varGamma (\textbf{s}, t_k) \right] )^4}. \end{aligned}$$

Detailed derivations are given in Appendix C.

**Fig. 4:**
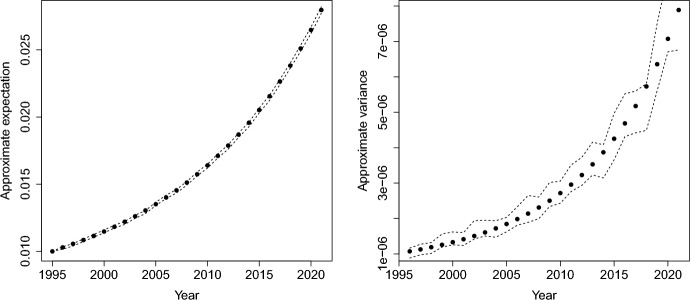
$95\%$ pointwise confidence intervals for the mean (left) and variance (right) of $\varGamma (\textbf{s}, t_k)^{-1}$ as a function of *k* when *m* is as in Table 1, $\alpha = 0.01$, $\gamma _0 = 100.0$ and $\sigma = 7.17$. The dots correspond to the approximations in Eq. (8) and Eq. (9)

The accuracy of the approximations can be assessed by comparing them to population estimates. Consider again the pore pressure values near the town of Slochteren listed in Table 1 and recall that the fitted regression variance is given by $\hat{\sigma }= 7.17$ (cf. Appendix A) and that $\varDelta =1$. To complete the Cox rate-and-state model, the parameters $\alpha$ and $\gamma _0$ must be specified. Setting, for example, $\gamma _0 = 100$ and $\alpha = 0.01$, the $95\%$ pointwise confidence intervals for the mean and variance are given in the left- and right-most panels of Fig. 4. In both cases, the sample size is $n=2,000$. It can be seen that the approximations are quite adequate. For details on the construction of the confidence intervals, see Appendix D.

## Parameter Estimation

The Cox process defined by Eq. (6) depends on several parameters: the regression parameters $\beta _i$ and $\sigma ^2$ used in the estimation of the pore pressure map, $\theta _1$, $\theta _2$, $\alpha$ and $\eta$. As the former parameters have already been estimated by the least squares approach (cf. Sect. 2 and Appendix A), denote the vector of remaining parameters by ${\boldsymbol{\zeta }} = (\theta _1, \theta _2, \alpha , \eta )$. Since the likelihood of a Cox process is intractable (Møller and Waagepetersen 2004), an estimating equations approach (Vaart 1998) can be used for $\boldsymbol{\zeta }$.

The unbiased estimating equation from Waagepetersen (2007) based on the gradient of the Poisson likelihood function is widely used in spatial statistics. However, this method cannot be applied directly since it assumes that the intensity function $\lambda$ is known analytically. For the Cox rate-and-state model, though, the intensity function, which reads

$$\begin{aligned} \lambda (\textbf{s}_i, t_j; \boldsymbol{\zeta }) = \alpha e^{ -\eta + \theta _1 + \theta _2 V(\textbf{s}_i,t_j)} {\mathbb E}_{\boldsymbol{\zeta }}\left[ \varGamma (\textbf{s}_i,t_j)^{-1} \right] , \end{aligned}$$

depends on the intractable expectation of the rate variable. Therefore, consider the modified estimating equation

10 $$\begin{aligned} \textbf{F}( \boldsymbol{\zeta }) = \sum _{(\textbf{s}_i, t_j)} \frac{\nabla h(\textbf{s}_i, t_j; \boldsymbol{\zeta })}{h(\textbf{s}_i, t_j; \boldsymbol{\zeta })} \left( N(\textbf{s}_i, t_j) - \widehat{\lambda (\textbf{s}_i, t_j; \boldsymbol{\zeta })} \varDelta \varDelta (\textbf{s}_i) \right) = 0, \end{aligned}$$

where the $\textbf{s}_i$ range through the cell representatives in $W_S$ and the $t_j$ indicate the time intervals,

$$\begin{aligned} h(\textbf{s}_i, t_j; \boldsymbol{\zeta }) = \frac{ e^{ \theta _1 + \theta _2 V(\textbf{s}_i, t_j ) } e^{ - \alpha m(\textbf{s}_i, t_j)} }{ e^\eta \varDelta \sum _{k=0}^{j-1} e^{ - \alpha m(\textbf{s}_i, t_k) } + e^{ - \alpha m(\textbf{s}_i, t_0) } }, \end{aligned}$$

and $\hat{\lambda }$ is an estimator for $\lambda$. Note that the function *h* is equal to the intensity function when there is no noise (i.e. $\sigma ^2 = 0$), in which case Eq. (10) reduces to Poisson likelihood estimation (Waagepetersen 2007). To estimate the intensity function, use

$$\begin{aligned} \widehat{\lambda (\textbf{s}_i, t_j; \boldsymbol{\zeta })} = e^{ \theta _1 + \theta _2 V(\textbf{s}_i, t_j ) } \frac{1}{L} \sum _{l=1}^L \frac{ e^{ - \alpha X_l(\textbf{s}_i, t_j )} }{ e^\eta \varDelta \sum _{k=0}^{j-1} e^{ - \alpha X_l(\textbf{s}_i, t_k) } + e^{ - \alpha X_l(\textbf{s}_i, t_0) } }. \end{aligned}$$

Here, $X_l$, $l=1, \dots , L$, are independent samples of $X = m + E$. Since

$$\begin{aligned} {\mathbb E}_{\boldsymbol{\zeta }} \left[ N(\textbf{s}_i, t_j) - \widehat{\lambda (\textbf{s}_i, t_j; \boldsymbol{\zeta })} \varDelta \varDelta (\textbf{s}_i) \right] = \lambda (\textbf{s}_i,t_j; \boldsymbol{\zeta }) \varDelta \varDelta (\textbf{s}_i) - \lambda (\textbf{s}_i,t_j; \boldsymbol{\zeta }) \varDelta \varDelta (\textbf{s}_i) = 0, \end{aligned}$$

Eq. (10) is unbiased. It can be solved numerically.

Returning to the Groningen data (cf. Sect. 2), recall from Lieshout and Baki (2024) that $\hat{\sigma }= 7.17$. Regarding the other parameters, $\hat{\eta }$ is effectively $-\infty$ (in view of the machine precision), $\hat{\theta }_1=-5.3$, $\hat{\theta }_2 = 9.7$ and $\hat{\alpha }= 0.0097$ using $L=1,000$ samples of *X* for $\hat{\lambda }$.

The quality of an estimating equation is expressed in terms of the covariance matrix $\varSigma _{\textbf{F}({\boldsymbol{\zeta }}_0)}$ of $\textbf{F}(\boldsymbol{\zeta }_0)$ under the true value $\boldsymbol{\zeta }_0$ of the parameter vector. However, since multiplying the left- and right-hand sides of Eq. (10) by the same constant does not alter the estimator but does affect the variance of the left-hand side, one needs to fix the scale. The conventional choice is to use $U(\boldsymbol{\zeta }_0) = -{\mathbb E}_{\boldsymbol{ \zeta }_0} \left[ J_{\textbf{F}}(\boldsymbol{ \zeta }_0) \right]$, the expectation of the negative Jacobian (Godambe 1985; Godambe and Heyde 2010) for this purpose. Doing so, the variance of the resulting scaled estimating equation is known as the inverse Godambe information matrix. See Appendix E for explicit expressions of $\textbf{F}(\boldsymbol{\zeta })$, for its Jacobian and for an asymptotic expression of the Godambe information matrix when the discretisation mesh goes to zero.

Under an appropriate asymptotic scheme, for example by letting the discretisation get finer and finer, and the observation window larger and larger, the inverse of the Godambe information matrix can be interpreted as the variance of $\hat{\boldsymbol{\zeta }}$. However, for the Groningen data, in view of the small earthquake counts and numerical stability considerations, the discretisation is rather coarse. Therefore it is better to use a parametric bootstrap approach (Vaart 1998) to obtain approximate confidence intervals, which are listed in Table 2. The details of the parametric bootstrap procedure are deferred to Appendix F.

**Table 2:** 95% confidence intervals for the components of the parameter vector ${\boldsymbol{\zeta }}$

Parameter	Confidence interval
$\theta _1$	$(-5.6, -5.1)$
$$\theta _2$$	(7.0, 12.8)
$$\alpha $$	(0.006, 0.01)
$\eta$	N/A

Note that the confidence interval for $\theta _2$ contains only strictly positive values, indicating that an increase in production leads to a higher earthquake hazard the next year.

**Fig. 5:**
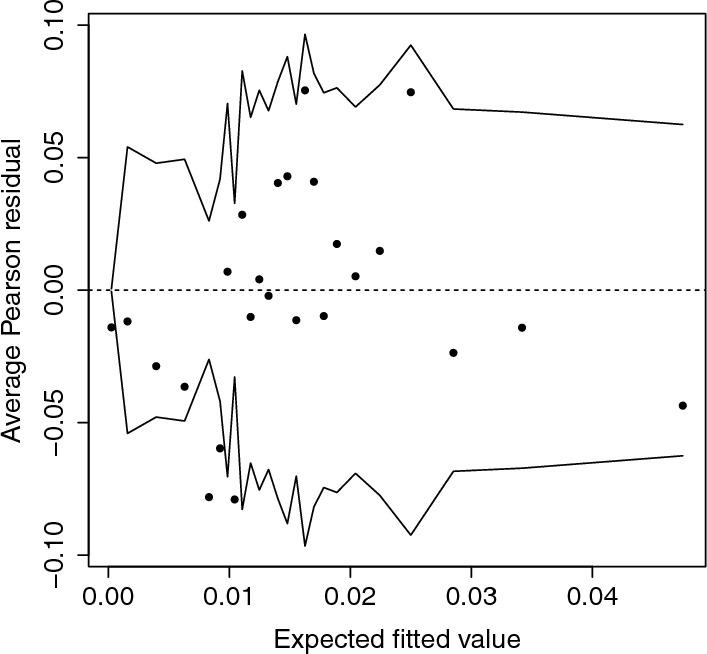
Average Pearson residual against expected fitted value for 25 bins for the model defined by Eq. (11). The grey lines correspond to two standard deviations bounds

Since $\exp (\hat{\eta }) = 0$, the fitted earthquake intensity function, which is given by

$$\begin{aligned} \lambda (s_i, t_j; \hat{\boldsymbol{\zeta }}) = \exp \left( \hat{\theta }_1 + \hat{\theta }_2 V(s_i, t_j) + \hat{\alpha }( m(s_i, t_0) - m(s_i, t_j) ) + \hat{\alpha }^2 \hat{\sigma }^2 \right) , \end{aligned}$$

can be evaluated explicitly. Therefore, model validation can be carried out by considering the Pearson residuals

$$\begin{aligned} \frac{ n(\textbf{s}_i, t_j) - \lambda (\textbf{s}_i, t_j; \hat{\boldsymbol{\zeta }}) \varDelta \varDelta (\textbf{s}_i) }{ \sqrt{ \lambda (\textbf{s}_i, t_j; \hat{\boldsymbol{\zeta }}) \varDelta \varDelta (\textbf{s}_i) + \lambda (\textbf{s}_i, t_j; \hat{\boldsymbol{\zeta }})^2 ( e^{2\hat{\alpha }^2\hat{\sigma }^2} - 1 ) \varDelta ^2 \varDelta (\textbf{s}_i)^2 } }, \end{aligned}$$

for $t_j > t_0$ and all $\textbf{s}_i$. Because the observed incidence counts $n(\textbf{s}_i, t_j)$ take only very small values, residual plots are not helpful in assessing the model fit. A better alternative is to divide the data into bins based on their fitted values and plot the average residuals against the average fitted value for each bin, as shown in Fig. 5. For most of the range, the average residual falls within two standard deviations; at around 0.01, the intensity is slightly overestimated.

To summarise, our final model is a log-Gaussian Cox process (Brix and Diggle 2001; Coles and Jones 1991; Møller et al. 1998) with driving random measure

11 $$\begin{aligned} \varDelta \varDelta (\textbf{s}_i) \exp \left( \hat{\theta }_1 + \hat{\theta }_2 V(\textbf{s}_i,t_j) + \hat{\alpha }\left\{ X(\textbf{s}_i, t_0) - X(\textbf{s}_i, t_j) \right\} \right) , \end{aligned}$$

where *X* is defined by Eq. (4).

## Monitoring Seismic Hazard

### Metropolis–Hastings Algorithm

Monitoring, assessing the current seismic hazard in light of past earthquakes, is based on the conditional distribution of $\varLambda$ or, equivalently, *E* given the recorded earthquakes. Write $\textbf{n}= (n(\textbf{s}_i, t_j))_{i, j}$ for the vector of observed earthquake counts $n(\textbf{s}_i, t_j)$ in the cells indexed by the $\textbf{s}_i$ and $t_j$. Then, upon ignoring all terms that do not depend on *E*, the log conditional likelihood reads

12 $$\begin{aligned} \log f( e(\textbf{s}_i, t_j)_{i,j} | \textbf{n})= &  - \sum _{(\textbf{s}_i, t_j)} \frac{ e(\textbf{s}_i, t_j)^2}{2 \sigma ^2} + \sum _{(\textbf{s}_i,t_j)} \left( n(\textbf{s}_i, t_j) \log \varLambda _{\textbf{e}}( \textbf{s}_i, t_j ) \right. \nonumber \\ &  \left. - \varDelta \varDelta (\textbf{s}_i) \varLambda _{\textbf{e}}( \textbf{s}_i, t_j ) \right) , \end{aligned}$$

where the $\textbf{s}_i$ range through the cell representatives in $W_S$ and the $t_j$ indicate the time intervals. Write $\varLambda _{\textbf{e}}$ to emphasise the dependence of the driving random measure on the realisation $\textbf{e}$ of *E*. The marginal conditional likelihood for fixed $\textbf{s}_i$ will be denoted by $f_{\textbf{s}_i}( e(\textbf{s}_i, t_j)_j | \textbf{n})$.

A Markov chain Monte Carlo technique, the Metropolis-adjusted Langevin algorithm (MALA) proposed by Besag (1994), is used to draw samples from Eq. (12). This algorithm is a Metropolis–Hastings sampler (Mengersen and Tweedie 1996) in which moves are proposed in the direction of the gradient of Eq. (12). It is important to observe that the independence of our model across the spatial domain allows for parallel implementation. Thus, for each spatial grid cell around $\textbf{s}$, repeat the steps in the following algorithm until the desired number of samples, *I*, is obtained.

#### [Style2 Style2]Algorithm 1

If the current state is $\textbf{e}(\textbf{s}) = (e(\textbf{s},t_0), \dots , e(\textbf{s}, t_m))$ and the earthquake count vector is $\textbf{n}$, then

1. Sample a realisation $\tilde{e}(\textbf{s},t_0), \dots , \tilde{e}(\textbf{s}, t_m)$ from independent normal distributions with variance *h* and, for $j>0$, mean
  $$\begin{aligned} \mu (\textbf{s}, t_j; \textbf{e}) = \left( 1 - \frac{h}{2\sigma ^2}\right) e(\textbf{s}, t_j) - \frac{h}{2} \alpha \left( n(\textbf{s}, t_j) - \varLambda _{\textbf{e}}(\textbf{s}, t_j) \varDelta \varDelta (\textbf{s}) \right) , \end{aligned}$$
  and
  $$\begin{aligned} \mu (\textbf{s}, t_0; \textbf{e}) = \left( 1 - \frac{h}{2\sigma ^2}\right) e(\textbf{s}, t_0) + \frac{h}{2} \alpha \sum _{i = 1}^{m} \left( n(s, t_i) - \varLambda _{\textbf{e}}(\textbf{s}, t_i) \varDelta \varDelta (\textbf{s}) \right) . \end{aligned}$$
2. Accept the new state with probability
  $$\begin{aligned} \frac{ f_{\textbf{s}}(\tilde{e}(\textbf{s}, t_0), \dots , \tilde{e}(\textbf{s}, t_m) | \textbf{n}) \exp \left( - \sum _{j=0}^m ( e(\textbf{s}, t_j) - \mu ({\textbf{s}, t_j}; {\tilde{\textbf{e}}}))^2 / (2h) \right) }{ f_{\textbf{s}}(e(\textbf{s}, t_0), \dots , e(\textbf{s}, t_m) | \textbf{n}) \exp \left( - \sum _{j=0}^m ( \tilde{e}(\textbf{s}, t_j) - \mu (\textbf{s}, t_j; \textbf{e}))^2 / (2h) \right) }. \end{aligned}$$

Since the proposals are governed by a normal distribution, which has a strictly positive probability density, by (Mengersen and Tweedie 1996, Lemma 1.1), the Markov chain generated by the MALA algorithm above is $f_{\textbf{s}}$-irreducible. Also, $f_{\textbf{s}}$ is a strictly positive probability density on ${\mathbb R}^m$, so by (Mengersen and Tweedie 1996, Lemma 1.2) the Markov chain is aperiodic. By construction, $f_{\textbf{s}}$ is an invariant measure. Thus the Markov chain converges in total variation from almost all initial states (Møller and Waagepetersen 2004, Proposition 7.7).

**Fig. 6:**
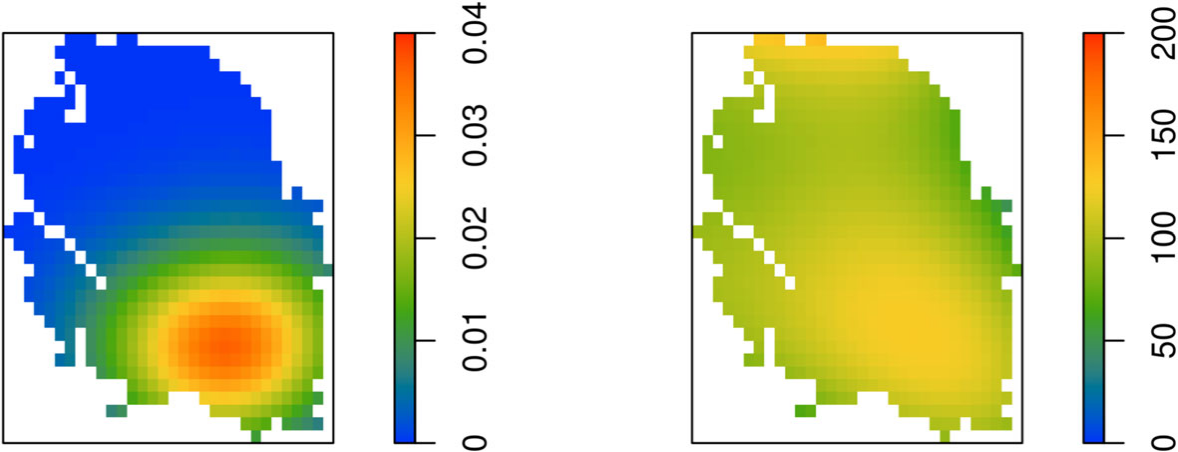
Left: Smoothed gas production over 2021 (in Nbcm for each grid cell). Right: Estimated pressure drop (in bara for each grid cell) from 1995 until 2022

### Results

Having obtained samples from the conditional distribution of $\varLambda$, the earthquake hazard can now be monitored. Monitoring targets the conditional distribution of counts in the time interval $[t_{m+1}, t_{m+1} + \varDelta ]$, that is, in the year 2022. For the Groningen data, recalling Eq. (11), these counts follow a Poisson distribution with intensity

$$\begin{aligned} \exp \left( \hat{\theta }_1 + \hat{\theta }_2 V(\textbf{s}, t_m + \varDelta ) + \hat{\alpha }\left\{ X_i (\textbf{s}, t_0 ) - m(\textbf{s}, t_m + \varDelta ) - E_i(\textbf{s}, t_m + \varDelta ) \right\} \right) \varDelta \varDelta (s), \end{aligned}$$

where the family $\left\{ X_i(\textbf{s},t_0) = m(\textbf{s}, t_0) + E_i(\textbf{s}, t_0) \right\} _{i=1, \dots , I}$ indexed by cell representatives $\textbf{s}$ use the samples $E_i(\textbf{s},t_0)$ from its conditional distribution given the counts generated by Algorithm 1 above, and where $E_i(\textbf{s}, t_m + \varDelta )$ is white noise with variance $\hat{\sigma }^2$. The parameter *h* in Algorithm 1 was set to $0.02\, \hat{\sigma }$, a burn-in period of 10, 000 steps for each spatial grid cell was used in combination with subsampling every 1, 000 steps until $I= 5,000$ realisations were obtained.

**Fig. 7:**
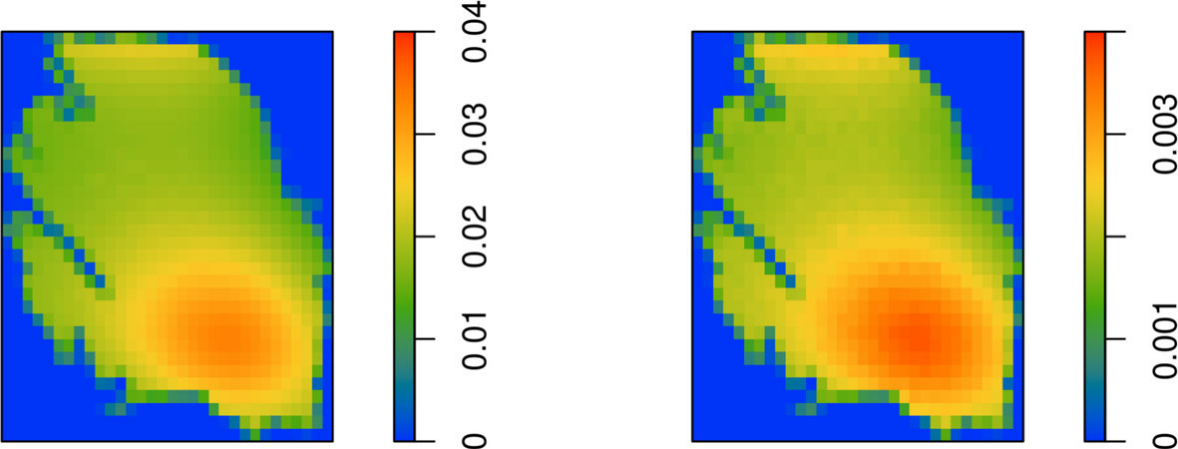
Left: Mean conditional number of earthquakes in 2022 (for each grid cell, sample size $I=5,000$). Right: Sample standard deviation of conditional number of earthquakes in 2022 (for each grid cell, sample size $I=5,000$)

Figure 6 plots the gas production in the preceding year 2021 and the estimated drop in pressure up to 2022. Note that the total volume of gas extracted was low, 6.48 Nbcm compared to over 50 Nbcm in 2013, and concentrated in the south of the gas field. As for the pressure, it can be seen that the estimated decrease in pressure is smallest in the western and eastern periphery. Because the wells in the south were taken into production earlier than those in the north, initially a larger drop in pressure was measured in the south. To reduce this imbalance somewhat, in the 1970s, 1980s and 1990s, extraction plans were adjusted so that the northern locations were preferred for production. However, in response to concerns following a major earthquake, the Dutch government imposed production caps on some northern clusters in 2014, which again emphasised the larger drop in pore pressure in the south. Additionally, the right panel of Fig. 6 shows a larger estimated fall in pressure in the far north offshore part of the field. Indeed, the observation well at Oldorp in the north-western corner of the field (cf. Fig. 2) is known to be atypical for the field: quite high values were observed in 1995 and there are very few recent measurements. See Jager and Visser (2017) for a more detailed description of the geology of the Groningen field.

The mean and standard deviation of the conditional intensity of earthquakes in each spatial cell around $\textbf{s}$ for the year 2022 are given in Fig. 7. Overall, the intensity of earthquakes is low. The area of increased risk due to gas extraction in the south of the field is tilted according to the pore pressure gradient, there is a smaller risk in the peripheral regions and the absence of production in the far north offsets the large drop in pressure. The standard deviation of the conditional distribution is highest in the large production region in the southeast and in the offshore northern region.

**Fig. 8:**
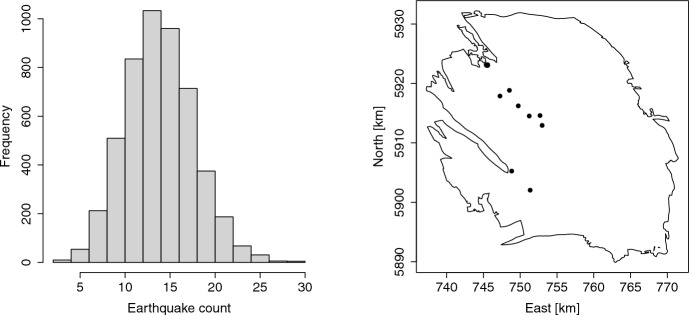
Histogram of a sample of size $I=5,000$ from the conditional distribution of the number of earthquakes of magnitude at least 1.5 in 2022 (left) and pattern of observed earthquakes of magnitude at least 1.5 in 2022 (right). The coordinates of the quakes are in the UTM system (zone 31, in kilometres)

The histogram of earthquake counts under the conditional distribution is given in the left panel of Fig. 8. For comparison, the pattern of the 12 recorded earthquakes of magnitude at least 1.5 that occurred in 2022 is shown in the right panel.

## Conclusion

This paper explored the seismic risk in the Groningen gas field by modifying the state-of-the-art rate-and-state model in two directions, namely allowing for noise in pore pressure measurements and explicitly taking into account gas production volumes. The first- and second-moment measures of the resulting Cox process were investigated and its parameters were estimated by a tailor-made estimating equation. Also, a Markov chain Monte-Carlo algorithm was constructed to monitor seismicity.

An important feature of our approach is that it is completely data-driven and does not rely on reservoir models. The advantage of working in a data-driven fashion is that uncertainty quantification is part of the toolbox; a drawback is that the accuracy depends on the quality of the data at hand. In our context, from the mid-1990s, the earthquake catalogue maintained by the Royal Netherlands Meteorological Office (KNMI 2022) is accurate. Production figures, too, are available at various public websites, but not always accurate. Therefore data provided directly by the production company Nederlandse Aardolie Maatschappij (NAM 2022) were used. Pore pressure measurements, however, are available only at wells and are quite sparse. Especially in recent years, there have not been many observations. In view of the fact that caps on production were put in place in 2014 and that production stopped altogether in 2024, which will likely affect the future drop in pore pressure, it would be wise to step up efforts in pore pressure measurements to improve the monitoring of seismic hazard.

The model can be extended in various directions. For instance, other explanatory variables, such as information on fault lines and subsidence, or other geological features of the field, could be taken into account. Also, spatially correlated random factors could be added to the model. For instance, one might take a spatio-temporal Ornstein–Uhlenbeck process (Brix and Diggle 2001), which, for $k=0, 1, \dots , m-1$ is defined by

$$\begin{aligned} U(\textbf{s}, t_{k+1}) = - \frac{\tau ^2}{2} ( 1 - e^{-\mu \varDelta } ) + e^{-\mu \varDelta } U(\textbf{s}, t_k ) + W(\textbf{s}, t_{k+1} ), \end{aligned}$$

where $\mu$ is a temporal decay parameter and where the vectors $W(\textbf{s}, t_{k+1})_{\textbf{s}}$ over the spatial locations $\textbf{s}$ are independent and normally distributed with mean zero and covariance matrix

$$\begin{aligned} \tau ^2 \left( 1 - e^{-2\mu \varDelta } \right) R, \end{aligned}$$

for some correlation matrix *R*, under the convention that $U(\textbf{s}, t_0) = W(\textbf{s},t_0) - {\tau ^2}/{2}$, where $W(\textbf{s},t_0)_s$ has covariance matrix $\tau ^2 R$, $\tau ^2 > 0$. From a theoretical point of view, an asymptotic theory for the estimating equation would be of interest.

Finally, one of us compared the data-driven approach in this paper with one that inputs the NAM reservoir model for the pore pressure (Baki 2024). In other words, the function *m* in Eq. (4) is replaced by a model based on geomechanical equations that incorporate the physical principles governing pressure changes in the reservoir. The conditional hazard maps for the geomechanical model are similar to the ones in this paper in overall appearance, with slightly more emphasis on the south. However, the residual variance after fitting the model parameters is smaller.

## Data Availability

The code is available from the authors on request.

## Appendix A: Estimated Pore Pressure Parameters

Let the pore pressure $X(\textbf{s},t)$, $\textbf{s}\in W_S$, $t\in W_T$ be a random variable that can be decomposed into a deterministic trend term $m((\textbf{s},t); \boldsymbol{\beta })$ and noise $E(\textbf{s},t)$ as

$$\begin{aligned} X(\textbf{s},t) = m((\textbf{s},t); \boldsymbol{\beta }) + E(\textbf{s},t). \end{aligned}$$

Assume that the measurement errors $E(\textbf{s},t)$ are independent and normally distributed with mean zero and variance $\sigma ^2$, and let $m(\textbf{s},t)$ be given by

$$\begin{aligned} m( (\textbf{s}, t); \boldsymbol{\beta })= &  \beta _1 + \beta _2 t + \beta _3 t^2 + \beta _4 (\textbf{s}-\textbf{s}_0)_1 + \beta _5 (\textbf{s}-\textbf{s}_0)_2 + \beta _6 (\textbf{s}-\textbf{s}_0)_1^2 \\ &  + \beta _7 (\textbf{s}-\textbf{s}_0)_1 (\textbf{s}-\textbf{s}_0)_2 + \beta _8 (\textbf{s}-\textbf{s}_0)_2^2 + \beta _9 (\textbf{s}-\textbf{s}_0)_1^3 \\ &  + \beta _{10} (\textbf{s}-\textbf{s}_0)_1^2 (\textbf{s}-\textbf{s}_0)_2 + \beta _{11} (\textbf{s}-\textbf{s}_0)_1 (\textbf{s}-\textbf{s}_0)_2^2 + \beta _{12} (\textbf{s}-\textbf{s}_0)_2^3 \\ &  + \beta _{13} (\textbf{s}-\textbf{s}_0)_1^4 + \beta _{14} (\textbf{s}-\textbf{s}_0)_1^3 (\textbf{s}-\textbf{s}_0)_2 + \beta _{15} (\textbf{s}-\textbf{s}_0)_1^2 (\textbf{s}-\textbf{s}_0)_2^2 \\ &  + \beta _{16} (\textbf{s}-\textbf{s}_0)_1 (\textbf{s}-\textbf{s}_0)_2^3 + \beta _{17} (\textbf{s}-\textbf{s}_0)_2^4 + \beta _{18} t (\textbf{s}-\textbf{s}_0)_1 + \beta _{19} t (\textbf{s}-\textbf{s}_0)_2 \\ &  + \beta _{20} t (\textbf{s}-\textbf{s}_0)_1^2 + \beta _{21} t (\textbf{s}-\textbf{s}_0)_1 (\textbf{s}-\textbf{s}_0)_2 + \beta _{22} t (\textbf{s}-\textbf{s}_0)_2^2 + \beta _{23} t (\textbf{s}-\textbf{s}_0)_1^3 \\ &  + \beta _{24} t (\textbf{s}-\textbf{s}_0)_1^2 (\textbf{s}-\textbf{s}_0)_2 + \beta _{25} t (\textbf{s}-\textbf{s}_0)_1 (\textbf{s}-\textbf{s}_0)_2^2 + \beta _{26} t (\textbf{s}-\textbf{s}_0)_2^3, \end{aligned}$$

where we centre the spatial locations at $\textbf{s}_0 = ( (s_0)_1, (s_0)_2) = (750, 5900)$ in the UTM system (zone 31, in kilometres).

The model is fitted sequentially by the least squares method: a first-order linear model in time is updated with a second-order term, first- up to fourth-order polynomials in space, and first- up to third-order space-time interaction terms. The second column in the ANOVA Table 3 lists the sum of squares for the coefficients in the first column, the third column quantifies the significance as the *p*-value of the F-test.

**Table 3:** ANOVA table for the pore pressure regression

	Sum Sq	Pr$(> F)$
$\beta _1 + \beta _2 t$	234,611	2.2e$-$16
$\beta _3 t^2$	1,976	6.085e$-$09
$\beta _4(\textbf{s}-\textbf{s}_0)_1 + \beta _5(\textbf{s}-\textbf{s}_0)_2$	15,261	2.2e$-$16
$\beta _6(\textbf{s}-\textbf{s}_0)^2_1 + \cdots + \beta _8 (\textbf{s}-\textbf{s}_0)^2_2$	58,675	2.2e$-$16
$\beta _9(\textbf{s}-\textbf{s}_0)^3_1 + \cdots + \beta _{12}(\textbf{s}-\textbf{s}_0)^3_2$	12,153	2.2e$-$16
$\beta _{13}(\textbf{s}-\textbf{s}_0)^4_1 + \cdots + \beta _{17}(\textbf{s}-\textbf{s}_0)^4_2$	25,106	2.2e$-$16
$\beta _{18} t (\textbf{s}-\textbf{s}_0)_1 + \beta _{19} t (\textbf{s}-\textbf{s}_0)_2$	1,502	2.189e$-$06
$\beta _{20} t (\textbf{s}-\textbf{s}_0)^2_1 + \cdots + \beta _{22} t (\textbf{s}-\textbf{s}_0)^2_2$	1,773	1.041e$-$06
$\beta _{23} t (\textbf{s}-\textbf{s}_0)^3_1 + \cdots + \beta _{26} t (\textbf{s}-\textbf{s}_0)^3_2$	1,148	0.0004642
Residual sum of squares	18,052	

The $95\%$ confidence intervals for the parameter vector $\boldsymbol{\beta }= (\beta _1, \dots , \beta _{26})$ are given in Table 4. The standard deviation of $E(\cdot , \cdot )$ is estimated at $\hat{\sigma }= 7.17$.

**Table 4:** Confidence intervals for the regression parameters

	Confidence interval		Confidence interval
$\beta _1$	(1.76e+02, 1.84e+02)	$\beta _2$	(-7.90e$-$03, -4.99e$-$03)
$\beta _3$	(-6.06e$-$07, -3.15e$-$07)	$\beta _4$	(-1.33e+01, -1.08e+01)
$\beta _5$	(-1.13e+01, -8.15e+00)	$\beta _6$	(1.71e+00, 2.04e+00)
$\beta _7$	(2.49e+00, 3.08e+00)	$\beta _8$	(9.47e$-$01, 1.34e+00)
$\beta _9$	(-1.22e$-$01, -9.57e$-$02)	$\beta _{10}$	(-2.59e$-$01, -2.14e$-$01)
$\beta _{11}$	(-1.91e$-$01, -1.50e$-$01)	$\beta _{12}$	(-5.84e$-$02, -3.95e$-$02)
$\beta _{13}$	(1.73e$-$03, 2.54e$-$03)	$\beta _{14}$	(5.28e$-$03, 6.46e$-$03)
$\beta _{15}$	(5.75e$-$03, 7.13e$-$03)	$\beta _{16}$	(2.44e$-$03, 3.30e$-$03)
$\beta _{17}$	(5.48e$-$04, 8.68e$-$04)	$\beta _{18}$	(-5.08e$-$04, -3.01e$-$05)
$\beta _{19}$	(-5.48e$-$04, 3.74e$-$05)	$\beta _{20}$	(-2.45e$-$05, 2.79e$-$05)
$\beta _{21}$	(-2.63e$-$05, 5.29e$-$05)	$\beta _{22}$	(9.19e$-$06, 5.93e$-$05)
$\beta _{23}$	(-3.05e$-$07, 1.59e$-$06)	$\beta _{24}$	(-1.01e$-$06, 1.71e$-$06)
$\beta _{25}$	(-1.54e$-$06, 1.10e$-$06)	$\beta _{26}$	(-1.51e$-$06, -3.22e$-$07)

## Appendix B: Moments of the State Variable

When *Z* is normally distributed with mean $\mu$ and variance $\sigma ^2$,

$$\begin{aligned} {\mathbb E}\left[ e^{t Z}\right] = \exp \left( \mu t + \sigma ^2 t^2 / 2 \right) , \end{aligned}$$

thus the formula for ${\mathbb E}\left[ \varGamma (\textbf{s}, t_k)\right]$ in Sect. 4 follows immediately. Moreover, for $0 < k \le l$,

13 $$\begin{aligned} \mathrm{{Cov}}( \varGamma (\textbf{s}, t_k), \varGamma (\textbf{s}, t_l) )= &  \alpha ^2 \varDelta ^2 \sum _{i=0}^{k-1} \sum _{j=0}^{l-1} \mathrm{{Cov}}\left( e^{\alpha \left( X(\textbf{s}, t_k ) - X(\textbf{s}, t_i ) \right) }, e^{\alpha \left( X(\textbf{s}, t_l ) - X(\textbf{s}, t_j ) \right) } \right) \nonumber \\+ &  \alpha \varDelta \gamma _0 \sum _{i=0}^{k-1} \mathrm{{Cov}}\left( e^{\alpha \left( X(\textbf{s}, t_k ) - X(\textbf{s}, t_i ) \right) }, e^{\alpha \left( X(\textbf{s}, t_l ) - X(\textbf{s}, t_0) \right) } \right) \nonumber \\+ &  \alpha \varDelta \gamma _0 \sum _{j=0}^{l-1} \mathrm{{Cov}}\left( e^{\alpha \left( X(\textbf{s}, t_k ) - X(\textbf{s}, t_0) \right) }, e^{\alpha \left( X(\textbf{s}, t_l ) - X(\textbf{s}, t_j) \right) } \right) \nonumber \\+ &  \gamma _0^2 \mathrm{{Cov}}\left( e^{\alpha \left( X(\textbf{s}, t_k ) - X(\textbf{s}, t_0) \right) }, e^{\alpha \left( X(\textbf{s}, t_l ) - X(\textbf{s}, t_0) \right) } \right) . \end{aligned}$$

First, consider the double sum over *i* and *j*. Because of the independence of the components of the random vector *E*, summands for which *k*, *l*, *i* and *j* are all different do not contribute. Therefore, for $0 < k = l$, the total contribution of the double sum is

$$\begin{aligned} \alpha ^2 \varDelta ^2 d^2 \sum _{i=0}^{k-1} f_{ki}^2 \left( d^2 - 1 \right) + \alpha ^2 \varDelta ^2 d^2 \sum _{i=0}^{k-1} f_{ki} \sum _{j\ne i; j=0}^{k-1} f_{kj} \left( d - 1 \right) . \end{aligned}$$

For $0< k < l$, the double sum contributes non-zero entries for $i=j$ (which cannot be equal to *k* or *l*) and for $i\ne j = k$. Their total contribution is

$$\begin{aligned} \alpha ^2 \varDelta ^2 d^2 \sum _{i=0}^{k-1} f_{ki} f_{li} \left( d - 1 \right) - \alpha ^2 \varDelta ^2 d^2 \sum _{i=0}^{k-1} f_{li} \left( 1 - \frac{1}{d} \right) . \end{aligned}$$

Next consider the two single sums on the right-hand side of Eq. (13). If $0 < k = l$, they are identical and each one is equal to

$$\begin{aligned} \alpha \varDelta \gamma _0 d^2 \left\{ f_{k0}^2 (d^2 - 1) + (d-1) \sum _{i=1}^{k-1} f_{ki} f_{k0} \right\} . \end{aligned}$$

In the case that $0< k< l$, the sum over *i* has a non-zero contribution only for $i=0$, and the sum over *j* has non-vanishing contributions for $j=0$ and $j=k$. Adding these up, one obtains

$$\begin{aligned} \alpha \varDelta \gamma _0 d^2 f_{l0} \left\{ 2 f_{k0} (d - 1) - \left( 1 - \frac{1}{d} \right) \right\} . \end{aligned}$$

Finally, the last term in the expression on the right-hand side of Eq. (13) reads $\gamma _0^2 f_{k0}^2$ $d^2 ( d^2-1 )$ for $0 < k = l$ and $\gamma _0^2 f_{k0}f_{l0} d^2 (d - 1 )$ when $0< k < l$. Equation. (7) now follows from tallying up the various contributions and recalling that $\gamma _0 = \alpha e^{-\eta }$. The variance of the state variable is obtained by taking $k=l$.

When the pore pressure increases, the state variables are non-negatively correlated. To see this, consider $0 \le i< k < l$. Then, if $\sigma ^2 = 0$ or $d f_{ki} \ge 1$,

$$\begin{aligned} \alpha ^2 \varDelta ^2 f_{li} \{ f_{ki} d^2 (d-1) - d ( d-1) \} \ge 0. \end{aligned}$$

The latter condition is equivalent to

$$\begin{aligned} \alpha ^2 \sigma ^2 + \alpha \left( m(\textbf{s}, t_k) - m(\textbf{s}, t_i) \right) \ge 0, \end{aligned}$$

which is implied by the assumption that *m* is increasing. By the same argument, for increasing pore pressure, $\alpha \varDelta \gamma _0 f_{l0} \{ f_{k0} d^2 (d-1) - d (d-1) \} \ge 0$ and an appeal to Eq. (7) completes the proof.

Next, suppose that the pore pressure decreases. For $0 \le i< k < l$, consider $\alpha ^2 \varDelta ^2 f_{li}$ $d (d-1) \{ d f_{ki} - 1 \}$. The term in between curly brackets is negative if and only if

$$\begin{aligned} \alpha \left( m(\textbf{s}, t_k ) - m(\textbf{s}, t_i) \right) + \alpha ^2 \sigma ^2 < 0. \end{aligned}$$

Since *m* is decreasing, if $\alpha \sigma ^2 < \min _i \left\{ m(\textbf{s}, t_i) - m(\textbf{s}, t_{i+1)}) \right\}$,

$$\begin{aligned} \alpha ^2 \sigma ^2 < \alpha \left( m(\textbf{s}, t_i) - m(\textbf{s}, t_{i+1}) \right) \le \alpha \left( m(\textbf{s}, t_i) - m(\textbf{s}, t_k) \right) , \end{aligned}$$

so that $\alpha ^2 \varDelta ^2 f_{li} d (d-1) \{ d f_{ki} - 1 \} \le 0$. Since the same argument can be used to show that $\alpha \varDelta \gamma _0 f_{l0} d (d-1) \{ d f_{k0} - 1 \} \le 0$,

$$\begin{aligned} \mathrm{{Cov}}( \varGamma (\textbf{s}, t_k), \varGamma (\textbf{s}, t_l) ) - (\alpha \varDelta \gamma _0 + \gamma _0^2) d^2 (d-1) f_{k0} f_{l0}= &  \\ \mathrm{{Cov}}( \varGamma (\textbf{s}, t_k), \varGamma (\textbf{s}, t_l) ) - \alpha ^2 e^{-\eta } ( \varDelta + e^{-\eta } ) d^2 (d-1) f_{k0} f_{l0}\le &  0. \end{aligned}$$

If $$\alpha \sigma ^2 > m(\textbf{s}, t_0)$$, then

$$\begin{aligned} \alpha ^2 \sigma ^2 > \alpha m(\textbf{s}, t_0) \ge \alpha m(\textbf{s}, t_i) \ge \alpha \left( m(\textbf{s}, t_i) - m(\textbf{s}, t_k) \right) . \end{aligned}$$

Therefore $\alpha ^2 \varDelta ^2 f_{li} d (d-1) \{ d f_{ki} - 1 \} \ge 0$. Similarly, $\alpha \varDelta \gamma _0 f_{l0} d (d-1) \{ d f_{k0} - 1 \} \ge 0.$ Consequently, $\mathrm{{Cov}}( \varGamma (\textbf{s}, t_k), \varGamma (\textbf{s}, t_l) ) \ge 0$.

## Appendix C: Approximate Moments of the Rate Variable

To approximate the expectation of $1/\varGamma (\textbf{s}, t_k)$, apply the delta method based on the following Taylor expansion around $x_0$ equal to the expectation of $\varGamma (\textbf{s}, t_k)$,

$$\begin{aligned} \frac{1}{x_0+h} \approx \frac{1}{x_0} - \frac{h}{x_0^2} + \frac{1}{2!} \frac{ 2h^2}{x_0^3}. \end{aligned}$$

Upon taking the expectation, one obtains that

$$\begin{aligned} {\mathbb E}\left[ \frac{1}{\varGamma (\textbf{s}, t_k)} \right]\approx &  \frac{1}{{\mathbb E}\left[ \varGamma (\textbf{s}, t_k)\right] } - {\mathbb E}\left[ \frac{\varGamma (\textbf{s}, t_k) - {\mathbb E}\left[ \varGamma (\textbf{s}, t_k)\right] }{ ({\mathbb E}\left[ \varGamma (\textbf{s}, t_k)\right] )^2 } \right] \\ &  + {\mathbb E}\left[ \frac{ ( \varGamma (\textbf{s}, t_k ) - {\mathbb E}\left[ \varGamma (\textbf{s}, t_k)\right] )^2}{ ({\mathbb E}\left[ \varGamma (\textbf{s}, t_k) \right] )^3} \right] . \end{aligned}$$

The middle term on the right-hand side is zero, and Eq. (8) follows.

The Taylor expansion of the function $(x,y) \mapsto 1/(xy)$ around the point $({\mathbb E}\left[ \varGamma (\textbf{s}, t_k)\right] ,$ ${\mathbb E}\left[ \varGamma (\textbf{s}, t_l)\right] )$ yields, up to second-order moments, the approximation

$$\begin{aligned} \frac{1}{\varGamma (\textbf{s}, t_k)} \frac{1}{\varGamma (\textbf{s}, t_l)}\approx &  \frac{1}{{\mathbb E}\left[ \varGamma (\textbf{s}, t_k)\right] } \frac{1}{{\mathbb E}\left[ \varGamma (\textbf{s}, t_l)\right] } \\ &  + \frac{ \left( \varGamma (\textbf{s}, t_k) - {\mathbb E}\left[ \varGamma (\textbf{s}, t_k) \right] \right) \, \left( \varGamma (\textbf{s}, t_l) - {\mathbb E}\left[ \varGamma (\textbf{s}, t_l) \right] \right) }{ ({\mathbb E}\left[ \varGamma (\textbf{s}, t_k)\right] )^2 ( {\mathbb E}\left[ \varGamma (\textbf{s}, t_l) \right] )^2} \\ &  - \frac{\varGamma (\textbf{s}, t_k) - {\mathbb E}\left[ \varGamma (\textbf{s}, t_k)\right] }{ ({\mathbb E}\left[ \varGamma (\textbf{s}, t_k) \right] )^2 {\mathbb E}\left[ \varGamma (\textbf{s}, t_l)\right] } - \frac{\varGamma (\textbf{s}, t_l) - {\mathbb E}\left[ \varGamma (\textbf{s}, t_l)\right] }{ ({\mathbb E}\left[ \varGamma (\textbf{s}, t_l)\right] )^2 {\mathbb E}\left[ \varGamma (\textbf{s}, t_k)\right] } \\ &  + \frac{ \left( \varGamma (\textbf{s}, t_k) - {\mathbb E}\left[ \varGamma (\textbf{s}, t_k)\right] \right) ^2 }{ ({\mathbb E}\left[ \varGamma (\textbf{s}, t_k)\right] )^3 {\mathbb E}\left[ \varGamma (\textbf{s}, t_l)\right] } + \frac{ \left( \varGamma (\textbf{s}, t_l) - {\mathbb E}\left[ \varGamma (\textbf{s}, t_l) \right] \right) ^2 }{{\mathbb E}\left[ \varGamma (\textbf{s}, t_k) \right] ( {\mathbb E}\left[ \varGamma (\textbf{s}, t_l)\right] )^3}. \end{aligned}$$

Finally, take expectations to obtain an approximation of the expected cross product of the rate.

## Appendix D: Calculation of Confidence Intervals for Moments

Let $Y_1, \dots , Y_n$ be an independent and identically distributed sample of some random variable with mean $\mu$ and variance $\sigma ^2$. Then, an approximate confidence interval for $\mu$ is

$$\begin{aligned} \left( \bar{Y}_n - \frac{S_n}{\sqrt{n}} \xi _{1-\alpha /2}, \quad \bar{Y}_n + \frac{S_n}{\sqrt{n}} \xi _{1-\alpha /2} \right) . \end{aligned}$$

An approximate confidence interval for $\sigma ^2$ reads

$$\begin{aligned} \left( \frac{S_n^2}{1 + \xi _{1-\alpha /2} \sqrt{(} \frac{2 \zeta }{n-1} ) }, \quad \frac{S_n^2}{1 - \xi _{1-\alpha /2} \sqrt{(} \frac{2 \zeta }{n-1} ) } \right) . \end{aligned}$$

Here $\bar{Y}_n$ and $S_n^2$ are the sample mean and variance, and $\xi _{1-\alpha /2}$ is the $(1-\alpha /2)$-quantile of the standard normal distribution. Furthermore, $\zeta = \frac{1}{2} ( \gamma _4-1 )$, where $\gamma _4$ denotes the ratio of the fourth central moment and the squared variance. Since $\gamma _4$ is unknown, estimate it by $\widehat{\gamma _4} = { n \sum (Y_i - m)^4 } / { ( (n-1)^2 S_n^4 ) }$, where *m* is the sample median (Curto 2023).

## Appendix E: Partial Derivatives for Parameter Estimation

Write, for $\textbf{s}\in W_S$ the centre of a cell with area $\varDelta (\textbf{s})$ and $t_k = t_0 + k \varDelta$,

$$\begin{aligned} S(\textbf{s},t_k; \boldsymbol{\zeta }) = e^\eta \varDelta \sum _{i=0}^{k-1} e^{-\alpha ( m(\textbf{s}, t_i) - m(\textbf{s}, t_k) ) } + e^{-\alpha ( m(\textbf{s}, t_0) - m(\textbf{s}, t_k) ) }. \end{aligned}$$

Let *h* be as in Sect. 6. Then $$ \frac{\partial }{\partial \theta _1} h(\textbf{s}, t_k; \boldsymbol{\zeta }) / { h(s, t_k; \boldsymbol{\zeta }) } $$ $$ = 1$$, $$ \frac{\partial }{\partial \theta _2} h(\textbf{s}, t_k; \boldsymbol{\zeta }) / { h(\textbf{s}, t_k; \boldsymbol{\zeta }) } = V(\textbf{s}, t_k) $$ and

$$\begin{aligned} \frac{ \frac{\partial }{\partial \alpha } h(\textbf{s}, t_k; \boldsymbol{\zeta }) }{ h(\textbf{s}, t_k; \boldsymbol{\zeta }) } = - \frac{ \frac{\partial }{\partial \alpha } S(\textbf{s}, t_k; \boldsymbol{\zeta })}{S(\textbf{s}, t_k; \boldsymbol{\zeta })}, \quad \frac{ \frac{\partial }{\partial \eta } h(\textbf{s}, t_k; \boldsymbol{\zeta }) }{ h(\textbf{s}, t_k; \boldsymbol{\zeta }) } = - \frac{ \frac{\partial }{\partial \eta } S(\textbf{s}, t_k; \boldsymbol{\zeta })}{S(\textbf{s}, t_k; \boldsymbol{\zeta })}. \end{aligned}$$

These partial derivatives define $\textbf{F}(\boldsymbol{\zeta })$ in Eq. (10).

The partial derivatives of $S(\cdot , \cdot ; \boldsymbol{\zeta })$ can be calculated recursively,

$$\begin{aligned} S(\textbf{s},t_k; \boldsymbol{\zeta })= &  \left( S(\textbf{s},t_{k-1}; \boldsymbol{\zeta }) + e^\eta \varDelta \right) e^{-\alpha (m(\textbf{s},t_{k-1})-m(\textbf{s},t_k))}, \\ \frac{\partial }{\partial \alpha } S(\textbf{s},t_k; \boldsymbol{\zeta })= &  \left( \frac{\partial }{\partial \alpha } S(\textbf{s},t_{k-1}; \boldsymbol{\zeta }) \right) e^{-\alpha (m(\textbf{s},t_{k-1})-m(\textbf{s},t_k))} \\ &  - (m(\textbf{s},t_{k-1})-m(\textbf{s},t_k) ) S(\textbf{s},t_k; \boldsymbol{\zeta }), \\ \frac{\partial }{\partial \eta } S(\textbf{s},t_k; \boldsymbol{\zeta })= &  \left( \frac{\partial }{\partial \eta } S(\textbf{s},t_{k-1}; \boldsymbol{\zeta }) + e^\eta \varDelta \right) e^{-\alpha (m(\textbf{s},t_{k-1})-m(\textbf{s},t_k))}, \\ \frac{\partial ^2}{\partial \alpha ^2} S(\textbf{s},t_k; \boldsymbol{\zeta })= &  \left( \frac{\partial ^2}{\partial \alpha ^2} S(\textbf{s},t_{k-1}; \boldsymbol{\zeta }) \right) e^{-\alpha (m(\textbf{s},t_{k-1})-m(\textbf{s},t_k))} \\ &  - \left( m(\textbf{s},t_{k-1})-m(\textbf{s},t_k) \right) \frac{\partial }{\partial \alpha } S(\textbf{s},t_k; \boldsymbol{\zeta }) \\ &  - \left( m(\textbf{s},t_{k-1})-m(\textbf{s},t_k) \right) \left( \frac{\partial }{\partial \alpha } S(\textbf{s},t_{k-1}; \boldsymbol{\zeta }) \right) e^{-\alpha (m(\textbf{s},t_{k-1})-m(\textbf{s},t_k))}, \\ \frac{\partial ^2}{\partial \alpha \partial \eta } S(\textbf{s},t_k; \boldsymbol{\zeta })= &  \left( \frac{\partial ^2}{\partial \alpha \partial \eta } S(\textbf{s},t_{k-1}; \boldsymbol{\zeta }) \right) e^{-\alpha (m(\textbf{s},t_{k-1})-m(\textbf{s},t_k))} \\ &  - \left( m(\textbf{s},t_{k-1})-m(\textbf{s},t_k) \right) \frac{\partial }{\partial \eta } S(\textbf{s},t_k; \boldsymbol{\zeta }), \end{aligned}$$

and $$\frac{\partial ^2}{\partial \eta ^2} S(\textbf{s},t_k; \boldsymbol{\zeta }) = \frac{\partial }{\partial \eta } S(\textbf{s},t_k; \boldsymbol{\zeta })$$.

Equation. (10) may be solved by the Newton method that iteratively solves, for $\boldsymbol{\zeta }_{n+1} -\boldsymbol{\zeta }_n$, the equation

$$\begin{aligned} J_{\textbf{F}}(\boldsymbol{\zeta }_n) ( \boldsymbol{\zeta }_{n+1} - \boldsymbol{\zeta }_n ) = - \textbf{F}(\boldsymbol{\zeta }_n). \end{aligned}$$

The Jacobian $J_{\textbf{F}}(\boldsymbol{\zeta })$ is a 4-by-4 matrix with components

$$\begin{aligned} J_{\textbf{F}}(\boldsymbol{\zeta })_{1,1}= &  - \sum _{(\textbf{s}_i,t_j)} \widehat{\lambda (\textbf{s}_i,t_j; \boldsymbol{\zeta })} \varDelta \varDelta (\textbf{s}_i), \\ J_{\textbf{F}}(\boldsymbol{\zeta })_{2,1}= &  - \sum _{(\textbf{s}_i,t_j)} V(\textbf{s}_i,t_j) \widehat{\lambda (\textbf{s}_i,t_j; \boldsymbol{\zeta })} \varDelta \varDelta (\textbf{s}_i), \\ J_{\textbf{F}}(\boldsymbol{\zeta })_{1,2}= &  - \sum _{(\textbf{s}_i,t_j)} V(\textbf{s}_i,t_j) \widehat{\lambda (\textbf{s}_i,t_j; \boldsymbol{\zeta })} \varDelta \varDelta (\textbf{s}_i), \\ J_{\textbf{F}}(\boldsymbol{\zeta })_{2,2}= &  - \sum _{(\textbf{s}_i,t_j)} V(\textbf{s}_i,t_j)^2 \, \widehat{\lambda (\textbf{s}_i,t_j; \boldsymbol{\zeta })} \varDelta \varDelta (\textbf{s}_i), \\ J_{\textbf{F}}(\boldsymbol{\zeta })_{1,3}= &  - \sum _{(\textbf{s}_i,t_j)} \frac{\partial \widehat{\lambda (\textbf{s}_i,t_j; \boldsymbol{\zeta })}}{\partial \alpha } \varDelta \varDelta (\textbf{s}_i), \\ J_{\textbf{F}}(\boldsymbol{\zeta })_{2,3}= &  - \sum _{(\textbf{s}_i,t_j)} V(\textbf{s}_i,t_j) \frac{\partial \widehat{\lambda (\textbf{s}_i,t_j; \boldsymbol{\zeta })}}{\partial \alpha } \varDelta \varDelta (\textbf{s}_i), \\ J_{\textbf{F}}(\boldsymbol{\zeta })_{1,4}= &  - \sum _{(\textbf{s}_i,t_j)} \frac{\partial \widehat{\lambda (\textbf{s}_i,t_j; \boldsymbol{\zeta })}}{\partial \eta } \varDelta \varDelta (\textbf{s}_i), \\ J_{\textbf{F}}(\boldsymbol{\zeta })_{2,4}= &  - \sum _{(\textbf{s}_i,t_j)} V(\textbf{s}_i,t_j) \frac{\partial \widehat{\lambda (\textbf{s}_i,t_j; \boldsymbol{\zeta })}}{\partial \eta } \varDelta \varDelta (\textbf{s}_i), \\ J_{\textbf{F}}(\boldsymbol{\zeta })_{3,1}= &  \sum _{(\textbf{s}_i,t_j)} \frac{\frac{\partial S(\textbf{s}_i,t_j; \boldsymbol{\zeta })}{\partial \alpha }}{S(\textbf{s}_i,t_j; \boldsymbol{\zeta })} \widehat{\lambda (\textbf{s}_i,t_j; \boldsymbol{\zeta })} \varDelta \varDelta (\textbf{s}_i), \\ J_{\textbf{F}}(\boldsymbol{\zeta })_{3,2}= &  \sum _{(\textbf{s}_i,t_j)} \frac{\frac{\partial S(\textbf{s}_i,t_j; \boldsymbol{\zeta })}{\partial \alpha }}{S(\textbf{s}_i,t_j; \boldsymbol{\zeta })} V(\textbf{s}_i,t_j) \widehat{\lambda (\textbf{s}_i,t_j; \boldsymbol{\zeta })} \varDelta \varDelta (\textbf{s}_i), \\ J_{\textbf{F}}(\boldsymbol{\zeta })_{4,1}= &  \sum _{(\textbf{s}_i,t_j)} \frac{\frac{\partial S(\textbf{s}_i,t_j; \boldsymbol{\zeta })}{\partial \eta }}{S(\textbf{s}_i,t_j; \boldsymbol{\zeta })} \widehat{\lambda (\textbf{s}_i,t_j; \boldsymbol{\zeta })} \varDelta \varDelta (\textbf{s}_i), \\ J_{\textbf{F}}(\boldsymbol{\zeta })_{4,2}= &  \sum _{(\textbf{s}_i,t_j)} \frac{\frac{\partial S(\textbf{s}_i,t_j; \boldsymbol{\zeta })}{\partial \eta }}{S(\textbf{s}_i,t_j; \boldsymbol{\zeta })} V(\textbf{s}_i,t_j) \widehat{\lambda (\textbf{s}_i,t_j; \boldsymbol{\zeta })} \varDelta \varDelta (\textbf{s}_i). \end{aligned}$$

In none of these do the counts play a role. They do for the last two terms in the third row,

$$\begin{aligned} &  \sum _{(\textbf{s}_i,t_j)} \left\{ \left( \frac{ \frac{\partial S(\textbf{s}_i,t_j; \boldsymbol{\zeta })}{\partial \alpha } }{S(\textbf{s}_i,t_j; \boldsymbol{\zeta })}\right) ^2 - \frac{ \frac{\partial ^2 S(\textbf{s}_i,t_j; \boldsymbol{\zeta })}{\partial \alpha ^2} }{S(\textbf{s}_i,t_j; \boldsymbol{\zeta })} \right\} \left( N(\textbf{s}_i,t_j) - \widehat{\lambda (\textbf{s}_i,t_j; \boldsymbol{\zeta })} \varDelta \varDelta (\textbf{s}_i) \right) + \\ &  \quad \sum _{(\textbf{s}_i,t_j)} \frac{ \frac{\partial S(\textbf{s}_i,t_j; \boldsymbol{\zeta })}{\partial \alpha }}{S(\textbf{s}_i,t_j; \boldsymbol{\zeta })} \frac{\partial \widehat{\lambda (\textbf{s}_i,t_j; \boldsymbol{\zeta })}}{\partial \alpha } \varDelta \varDelta (\textbf{s}_i), \end{aligned}$$

respectively,

$$\begin{aligned} &  \sum _{(\textbf{s}_i,t_j)} \left\{ \frac{ \frac{\partial S(\textbf{s}_i,t_j; \boldsymbol{\zeta })}{\partial \alpha } }{S(\textbf{s}_i,t_j; \boldsymbol{\zeta })} \frac{ \frac{\partial S(\textbf{s}_i,t_j; \boldsymbol{\zeta }) }{\partial \eta } }{S(\textbf{s}_i,t_j; \boldsymbol{\zeta })} - \frac{ \frac{\partial ^2 S(\textbf{s}_i,t_j; \boldsymbol{\zeta })}{\partial \alpha \partial \eta } }{S(\textbf{s}_i,t_j; \boldsymbol{\zeta })} \right\} \left( N(\textbf{s}_i,t_j) - \widehat{\lambda (\textbf{s}_i,t_j;\boldsymbol{\zeta })} \varDelta \varDelta (\textbf{s}_i) \right) + \\ &  \quad \sum _{(\textbf{s}_i,t_j)} \frac{ \frac{\partial S(\textbf{s}_i,t_j; \boldsymbol{\zeta })}{\partial \alpha }}{S(s_i,t_j; \boldsymbol{\zeta })} \frac{\partial \widehat{\lambda (\textbf{s}_i,t_j; \boldsymbol{\zeta })}}{\partial \eta } \varDelta \varDelta (\textbf{s}_i). \end{aligned}$$

Finally, the two last entries of the fourth row are

$$\begin{aligned} &  \sum _{(\textbf{s}_i,t_j)} \left\{ \frac{ \frac{\partial S(\textbf{s}_i,t_j; \boldsymbol{\zeta })}{\partial \alpha } }{S(\textbf{s}_i,t_j; \boldsymbol{\zeta })} \frac{ \frac{\partial S(\textbf{s}_i,t_j; \boldsymbol{\zeta }) }{\partial \eta } }{S(\textbf{s}_i,t_j; \boldsymbol{\zeta })} - \frac{ \frac{\partial ^2 S(\textbf{s}_i,t_j; \boldsymbol{\zeta })}{\partial \alpha \partial \eta } }{S(\textbf{s}_i,t_j; \boldsymbol{\zeta })} \right\} \left( N(\textbf{s}_i,t_j) - \widehat{\lambda (\textbf{s}_i,t_j; \boldsymbol{\zeta })} \varDelta \varDelta (\textbf{s}_i) \right) + \\ &  \quad \sum _{(\textbf{s}_i,t_j)} \frac{ \frac{\partial S(\textbf{s}_i,t_j; \boldsymbol{\zeta })}{\partial \eta }}{S(\textbf{s}_i,t_j; \boldsymbol{\zeta })} \frac{\partial \widehat{\lambda (\textbf{s}_i,t_j; \boldsymbol{\zeta })}}{\partial \alpha } \varDelta \varDelta (\textbf{s}_i), \end{aligned}$$

and

$$\begin{aligned} &  \sum _{(\textbf{s}_i,t_j)} \left\{ \left( \frac{ \frac{\partial S(\textbf{s}_i,t_j; \boldsymbol{\zeta }) }{\partial \eta } }{S(\textbf{s}_i,t_j; \boldsymbol{\zeta })}\right) ^2 - \frac{ \frac{\partial ^2 S(\textbf{s}_i,t_j; \boldsymbol{\zeta })}{\partial \eta ^2} }{S(\textbf{s}_i,t_j; \boldsymbol{\zeta })} \right\} \left( N(\textbf{s}_i,t_j) - \widehat{\lambda (\textbf{s}_i,t_j; \boldsymbol{\zeta })} \varDelta \varDelta (\textbf{s}_i) \right) + \\ &  \quad \sum _{(\textbf{s}_i,t_j)} \frac{ \frac{\partial S(\textbf{s}_i,t_j; \boldsymbol{\zeta })}{\partial \eta }}{S(\textbf{s}_i,t_j; \boldsymbol{\zeta })} \frac{\partial \widehat{\lambda (\textbf{s}_i,t_j; \boldsymbol{\zeta })}}{\partial \eta } \varDelta \varDelta (\textbf{s}_i). \end{aligned}$$

Note that the Jacobian is not symmetric, as it does not necessarily correspond to a likelihood!

A recursive formula for the partial derivatives of $S(\cdot , \cdot ; \boldsymbol{\zeta })$ is given above. It remains to calculate the partial derivatives of $\widehat{\lambda (\cdot , \cdot ; \boldsymbol{\zeta })}$. Those with respect to $\theta _1$ and $\theta _2$ are, respectively, $\widehat{\lambda (\cdot , \cdot ; \boldsymbol{\zeta })}$ and $V \widehat{\lambda (\cdot , \cdot ; \boldsymbol{\zeta })}$. Furthermore,

$$\begin{aligned}&\frac{\partial \widehat{\lambda (\textbf{s},t_k; \boldsymbol{\zeta })}}{\partial \alpha } = e^{\theta _1 + \theta _2 {V}(\textbf{s},t_k)} \frac{1}{L} \sum _{l=1}^L \frac{-\frac{\partial }{\partial \alpha } S_{X_l}(\textbf{s},t_k; \boldsymbol{\zeta })}{S_{X_l}(\textbf{s},t_k; \boldsymbol{\zeta })^2},\\&\frac{\partial \widehat{\lambda (\textbf{s},t_k; \boldsymbol{\zeta })}}{\partial \eta } = e^{\theta _1 + \theta _2 {V}(\textbf{s},t_k)} \frac{1}{L}\sum _{l=1}^L \frac{-\frac{\partial }{\partial \eta } S_{X_l}(\textbf{s},t_k; \boldsymbol{\zeta })}{S_{X_l}(\textbf{s},t_k; \boldsymbol{\zeta })^2}, \end{aligned}$$

with

$$\begin{aligned} S_{X_l} (\textbf{s}, t_k; \boldsymbol{\zeta })&= e^\eta \varDelta \sum _{i=0}^{k-1} e^{-\alpha ( {X_l} (\textbf{s},t_i) - {X_l} (\textbf{s}, t_k))} + e^{-\alpha ( {X_l} (\textbf{s},t_0) - {X_l} (\textbf{s},t_k) ) }, \\ \frac{\partial }{\partial \alpha }S_{X_l}(\textbf{s},t_k; \boldsymbol{\zeta })&= - (X_l(\textbf{s}, t_0) - X_l(\textbf{s},t_k)) e^{- \alpha (X_l(\textbf{s}, t_0) - X_l(\textbf{s},t_k))} \\&- e^{\eta }\varDelta \sum _{i=0}^{k-1} (X_l(\textbf{s},t_i) - X_l(\textbf{s},t_k)) e^{-\alpha (X_l(\textbf{s},t_i) - X_l(\textbf{s},t_k))}, \\ \frac{\partial }{\partial \eta }S_{X_l}(\textbf{s},t_k; \boldsymbol{\zeta })&= e^{\eta }\varDelta \sum _{i=0}^{k-1} e^{-\alpha (X_l(\textbf{s},t_i) - X_l(\textbf{s},t_k))}. \end{aligned}$$

The Godambe information matrix of Eq. (10) with $e^\eta = 0$ and remaining parameter vector $\boldsymbol{\zeta }= ( \theta _1, \theta _2, \alpha )$ is of the form $U(\boldsymbol{\zeta })^\top \varSigma _{\textbf{F}}(\boldsymbol{\zeta })^{-1} U(\boldsymbol{\zeta })$. In the limit upon letting $\varDelta$ and all $\varDelta (\textbf{s})$ go to zero, the first two columns of $U(\boldsymbol{\zeta })$ read

$$\begin{aligned} \left[ \begin{array}{l} \int _{W_S\times W_T} \lambda (\textbf{s},t; \boldsymbol{\zeta }) d\textbf{s} dt \\ \int _{W_S\times W_T} V(\textbf{s},t) \lambda (\textbf{s},t; \boldsymbol{\zeta }) d\textbf{s} dt \\ \int _{W_S\times W_T} ( m(\textbf{s}, t_0) - m(\textbf{s},t) ) \lambda (\textbf{s},t; \boldsymbol{\zeta }) d\textbf{s} dt \end{array} \right] , \end{aligned}$$

respectively

$$\begin{aligned} \left[ \begin{array}{l} \int _{W_S\times W_T} V(\textbf{s},t) \lambda (\textbf{s},t; \boldsymbol{\zeta }) d\textbf{s} dt \\ \int _{W_S\times W_T} V(\textbf{s},t)^2 \lambda (\textbf{s},t; \boldsymbol{\zeta }) d\textbf{s} dt \\ \int _{W_S\times W_T} ( m(\textbf{s}, t_0) - m(\textbf{s},t) ) V(\textbf{s},t) \lambda (\textbf{s},t; \boldsymbol{\zeta }) d\textbf{s} dt \end{array} \right] , \end{aligned}$$

where

$$\begin{aligned} \lambda (\textbf{s},t; \boldsymbol{\zeta }) = \exp \left( \theta _1 + \theta _2 V(\textbf{s},t) + \alpha ( m(\textbf{s}, t_0) - m(\textbf{s},t) ) + \alpha ^2 \sigma ^2 \right) , \quad t > t_0. \end{aligned}$$

The last column of $U(\boldsymbol{\zeta })$ is

$$\begin{aligned} \left[ \begin{array}{l} \int _{W_S\times W_T} l(\textbf{s},t; \boldsymbol{\zeta }) d\textbf{s} dt \\ \int _{W_S\times W_T} V(\textbf{s},t) l(s,t; \boldsymbol{\zeta }) d\textbf{s} dt \\ \int _{W_S\times W_T} ( m(\textbf{s}, t_0) - m(\textbf{s},t) ) l(s,t; \boldsymbol{\zeta }) d\textbf{s} dt \end{array} \right] , \end{aligned}$$

where

$$\begin{aligned} l(\textbf{s},t; \boldsymbol{\zeta }) = {\mathbb E}_{\boldsymbol{\zeta }} \left[ (X(\textbf{s}, t_0) - X(\textbf{s},t)) e^{ \theta _1 + \theta _2 V(\textbf{s},t) } e^{\alpha ( X(\textbf{s},t_0) - X(\textbf{s},t) )} \right] . \end{aligned}$$

The first two columns of the matrix $\varSigma _{\textbf{F}}(\boldsymbol{\zeta })$ are identical to those of $U(\boldsymbol{\zeta })$. Its last column reads

$$\begin{aligned} \left[ \begin{array}{l} \int _{W_S\times W_T} ( m(\textbf{s}, t_0) - m(\textbf{s},t) ) \lambda (\textbf{s},t; \boldsymbol{\zeta }) d\textbf{s} dt \\ \int _{W_S\times W_T} ( m(\textbf{s}, t_0) - m(\textbf{s},t) ) V(\textbf{s},t) \lambda (\textbf{s},t; \boldsymbol{\zeta }) d\textbf{s} dt \\ \int _{W_S\times W_T} ( m(\textbf{s}, t_0) - m(\textbf{s},t) )^2 \lambda (\textbf{s},t; \boldsymbol{\zeta }) d\textbf{s} dt \end{array} \right] . \end{aligned}$$

## Appendix F: Calculation of Bootstrap Confidence Intervals

The parametric bootstrap used in Sect. 6 proceeds as follows. For $k=1, \dots , 2,000$,

- under parameter values $\hat{ \boldsymbol{\zeta }}$, generate a realisation of the random vector that contains the $\varLambda (\textbf{s}_i, t_j)$ for all $(\textbf{s}_i, t_j)$,
- generate a realisation of the counts $N(\textbf{s}_i, t_j)$ from independent Poisson distributions with means $\varLambda (\textbf{s}_i, t_j) \varDelta \varDelta (\textbf{s}_i)$;
- estimate $\boldsymbol{\zeta }$ based on the counts $N(\textbf{s}_i, t_j)$ and denote the result by $\hat{\boldsymbol{\zeta }_k}$.

Next, write $\boldsymbol{\zeta }^*_k = \hat{ \boldsymbol{\zeta }_k} - \hat{ \boldsymbol{\zeta }}$, $k=1, \dots , 2,000$, for the bootstrap difference vectors. Then calculate the $2.5\%$ and $97.5\%$ quantiles of the components of $\boldsymbol{\zeta }^*_k$ and collect them in $\boldsymbol{\xi }_{2.5}$ and $\boldsymbol{\xi }_{97.5}$. The components of $( \hat{\boldsymbol{\zeta }} - \boldsymbol{\xi }_{97.5}, \hat{\boldsymbol{\zeta }} - \boldsymbol{\xi }_{2.5} )$ form bootstrap confidence intervals at level $95\%$.

## Footnotes

1 Source: Mr Rob van Eijs from Shell.

## References

[CR1] Baki Z (2024) Incorporation of NAM’s dynamic reservoir model into Cox rate-and-state model for monitoring of earthquakes in the Groningen gas field, arXiv:2409.18837

[CR2] Baki Z, Lieshout MNM van (2022) The influence of gas production on seismicity in the Groningen field. In: Proceedings of the 10th international workshop on spatio-temporal modelling METMA X, pp 163–167

[CR3] Besag J (1994) Discussion to Grenander and Miller, representations of knowledge in complex systems. J Roy Stat Soc B56:549–603

[CR4] Boitz N, Langenbruch C, Shapiro SA (2024) Production-induced seismicity indicates a low risk of strong earthquakes in the Groningen gas field. Nat Commun 15:328 38184655 10.1038/s41467-023-44485-4 PMC10771524

[CR5] Brix A, Diggle PJ (2001) Spatiotemporal prediction for log-Gaussian Cox processes. J Roy Stat Soc B63:823–841

[CR6] Candela T et al (2019) Depletion-induced seismicity at the Groningen gas field: Coulomb rate-and-state models including differential compaction effect. J Geophys Res Solid Earth 124:7081–7104

[CR7] Chiu SN, Stoyan D, Kendall WS, Mecke J (2013) Stochastic geometry and its applications, 3rd edn. Wiley, Chichester

[CR8] Coles P, Jones B (1991) A lognormal model for the cosmological mass distribution. Mon Not R Astron Soc 248:1–13

[CR9] Curto JD (2023) Confidence intervals for means and variances of non-normal distributions. Commun Stat Simul Comput 52:4414–4430

[CR10] Dempsey D, Suckale J (2017) Physics-based forecasting of induced seismicity at Groningen gas field. Neth Geophys Res Lett 22:7773–7782

[CR11] Godambe VP (1985) The foundations of finite sample estimation in stochastic processes. Biometrika 72:419–428

[CR12] Godambe VP, Heyde CC (2010) Quasi-likelihood and optimal estimation. In: Heyde CC (ed) Selected works. Springer, Berlin, pp 386–399

[CR13] Jager J de, Visser C (2017) Geology of the Groningen field-an overview. Neth J Geosci 96:3–15

[CR14] Kűhn D, Hainzl S, Dahm T, Richter G, Vera Rodriguez I (2022) A review of source models to further the understanding of the seismicity of the Groningen field. Neth J Geosci 101:e11

[CR15] Lieshout MNM van, Baki Z (2024) Exploring seismic hazard in the Groningen gas field using adaptive kernel smoothing. Math Geosci 56:1185–1206

[CR16] Mengersen KL, Tweedie RL (1996) Rates of convergence of the Hastings and Metropolis algorithms. Ann Stat 24:101–121

[CR17] Møller J, Waagepetersen RP (2004) Statistical inference and simulation for spatial point processes. Chapman & Hall, Chichester

[CR18] Møller J, Syversveen AR, Waagepetersen RP (1998) Log Gaussian Cox processes. Scand J Stat 25:451–482

[CR19] nam-feitenencijfers.data-app.html/gasdruk.html, downloaded April 2022 (2022)

[CR20] Richter G, Hainzl S, Dahm T, Zőller G (2020) Stress-based statistical modeling of the induced seismicity at the Groningen gas field. Neth Environ Earth Sci 79:252

[CR21] Utsu T, Ogata Y, Matsu’ura RS (1995) The centenary of the Omori formula for a decay law of aftershock activity. J Phys Earth 43:1–33

[CR22] Vaart AW van der (1998) Asymptotic statistics. Cambridge University Press

[CR23] Waagepetersen RP (2007) An estimating function approach to inference for inhomogeneous Neyman-Scott processes. Biometrics 63:252–258 17447951 10.1111/j.1541-0420.2006.00667.x

[CR24] www.knmi.nl/kennis-en-datacentrum/dataset/aardbevingscatalogus, downloaded April 2022 (2022)

[CR25] www.nlog.nl/bestanden-interactieve-kaart, downloaded April 2022 (2022)

